# Optimal bispectral index level of sedation and cerebral oximetry in traumatic brain injury: a non-invasive individualized approach in critical care?

**DOI:** 10.1186/s40635-022-00460-9

**Published:** 2022-08-13

**Authors:** Logan Froese, Alwyn Gomez, Amanjyot Singh Sainbhi, Carleen Batson, Trevor Slack, Kevin Y. Stein, Francois Mathieu, Frederick A. Zeiler

**Affiliations:** 1 Biomedical Engineering, Price Faculty of Engineering, University of Manitoba, Winnipeg, Canada; 2 Section of Neurosurgery, Department of Surgery, Rady Faculty of Health Sciences, University of Manitoba, Winnipeg, MB Canada; 3 Department of Human Anatomy and Cell Science, Rady Faculty of Health Sciences, University of Manitoba, Winnipeg, MB Canada; 4 Interdepartmental Division of Critical Care, Department of Medicine, University of Toronto, Toronto, Canada; 5 Department of Clinical Neuroscience, Karolinska Institutet, Stockholm, Sweden; 6 Division of Anaesthesia, Department of Medicine, Addenbrooke’s Hospital, University of Cambridge, Cambridge, UK

**Keywords:** Cerebral oximetry index, Cerebrovascular reactivity, Depth of sedation, Optimal bispectral index, Traumatic brain injury

## Abstract

**Background:**

Impaired cerebral autoregulation has been linked with worse outcomes, with literature suggesting that current therapy guidelines fail to significantly impact cerebrovascular reactivity. The cerebral oximetry index (COx_a) is a surrogate measure of cerebrovascular reactivity which can in theory be obtained non-invasively using regional brain tissue oxygen saturation and arterial blood pressure. The goal of this study was to assess the relationship between objectively measured depth of sedation through BIS and autoregulatory capacity measured through COx_a.

**Methods:**

In a prospectively maintained observational study, we collected continuous regional brain tissue oxygen saturation, intracranial pressure, arterial blood pressure and BIS in traumatic brain injury patients. COx_a was obtained using the Pearson’s correlation between regional brain tissue oxygen saturation and arterial blood pressure and ranges from − 1 to 1 with higher values indicating impairment of cerebrovascular reactivity. Using BIS values and COx_a, a curve-fitting method was applied to determine the minimum value for the COx_a. The associated BIS value with the minimum COx_a is called BISopt. This BISopt was both visually and algorithmically determined, which were compared and assessed over the whole dataset.

**Results:**

Of the 42 patients, we observed that most had a parabolic relationship between BIS and COx_a. This suggests a potential “optimal” depth of sedation where COx_a is the most intact. Furthermore, when comparing the BISopt algorithm with visual inspection of BISopt, we obtained similar results. Finally, BISopt % yield (determined algorithmically) appeared to be independent from any individual sedative or vasopressor agent, and there was agreement between BISopt found with COx_a and the pressure reactivity index (another surrogate for cerebrovascular reactivity).

**Conclusions:**

This study suggests that COx_a is capable of detecting disruption in cerebrovascular reactivity which occurs with over-/under-sedation, utilizing a non-invasive measure of determination and assessment. This technique may carry implications for tailoring sedation in patients, focusing on individualized neuroprotection.

**Supplementary Information:**

The online version contains supplementary material available at 10.1186/s40635-022-00460-9.

## Background

Patients with traumatic brain injury (TBI) requiring admission to the intensive care unit frequently show significant impairment in cerebrovascular autoregulation. Despite mounting evidence that cerebral autoregulatory failure is associated with poor outcome in TBI [1–5] and the emerging availability of individualized optimal cerebrovascular reactivity (a surrogate measure for cerebrovascular autoregulation) targets through the use of pressure reactivity index (PRx) and cerebral perfusion pressure (CPP) [6–8], current guideline-based therapies have demonstrated a limited impact on cerebrovascular reactivity [1, 9–12].

Our group has recently demonstrated an association between individualized depth of sedation (as measured through the bispectral index; BIS) and PRx [13, 14]. This indicates that there is potentially an optimal sedation depth, with regard to cerebrovascular reactivity, that can be attained during TBI care. This individualized sedation target, called BISopt, offers a potential avenue for true personalized medicine in moderate/severe TBI patients, focusing on the sedation target where cerebrovascular reactivity is the most intact. Our previous work has demonstrated that BISopt, determined using PRx, can be continuously derived at the bedside and varies between patients and over time [14]. Furthermore, we highlighted the impact of both over-sedation and under-sedation in this population, finding that both lead to worse cerebrovascular reactivity and potentially expose patients to ongoing secondary brain injuries [14].

Aside from critically ill TBI populations, the BISopt concept is attractive to the wider general ICU population, where various studies have documented the association between sedation exposure and poor neurologic and cognitive outcomes in survivors [9, 15–18]. Such general ICU populations have been shown to exhibit impaired cerebrovascular reactivity throughout the acute phase of care, which may in part explain the long-term cognitive deficits seen in such populations [16–21]. The BISopt personalized sedation target caries the potential to reduce secondary brain damage related to sedation-mediated impaired cerebrovascular reactivity [13, 14, 16–18]. Expansion of this technique to non-TBI populations is primarily limited by the need to continuously calculate PRx prior to deriving the BISopt, a process that requires invasive intracranial pressure (ICP) monitoring.

Methods of determining cerebrovascular reactivity focus on the slow wave vasogenic changes between the systemic and the intracranial circulatory system [7, 10, 22, 23]. Currently, the most utilized method is the PRx which assesses the slow wave changes in ICP and arterial blood pressure (ABP); however, in order to continuously record ICP an invasive intra-parenchymal pressure probe is implanted in the patient [7, 10, 22, 24]. Recent work has established that the cerebral oximetry index (COx_a), which assesses the slow wave vasogenic changes between near-infrared spectroscopy (NIRS) based regional brain tissue oxygenation (rSO_2_) and ABP, is comparable to PRx [25–30]. Similarly, the COx_a indices have been shown in pre-clinical literature to measure aspects of the Lassen autoregulatory curve [31–36]. This non-invasive measure of cerebrovascular reactivity determination opens the potential to evaluate cerebral autoregulation in non-TBI cohorts, and explore personalized cerebral physiologic targets focused on neuroprotection in such populations.

It remains unknown if the BISopt metrics can be derived using the relationship between non-invasive COx_a and BIS. Thus, the goal of this study is to evaluate the relationship between depth of sedation as determined by the BIS and COx_a, highlighting: (A) the presence of this novel BISopt target, and (B) preliminary attempts at continuous derivation of BISopt using COx_a.

## Methods

### Patient population

From September of 2020 to February of 2022, moderate/severe TBI patients were prospectively recruited to this observational study. All patients underwent BIS, NIRS, ICP and ABP monitoring during their ICU care. All patients received ICP/CPP directed treatment in accordance with the brain trauma foundation guidelines and Seattle International Severe Traumatic Brain Injury Consensus Conference, including treatment to target ICP and CPP thresholds [9, 37]. Thus, all sedation was administered in accordance with such guidelines, with the aim to minimize sedation based on what ICP/CPP would allow during the acute phase of care. From these patients, those with a frontal subgaleal hematoma or frontal lobe contusion were excluded as these may interfere with BIS and NIRS recordings [38]. Finally, all patients were mechanically ventilated during the recording of the described physiology.

### Ethical considerations

Data were collected following full approval by the University of Manitoba Health Research Ethics Board (H2017:181, H2017:188, B2018:103 and B2019:065) and the Health Sciences Centre Research Impact Committee (R2019:072). All patients had informed consent obtained as part of B2018:103.

### Data collection

As part of the prospective TBI database in Winnipeg, all patient demographics, admission characteristics and treatments (including pharmacologic information) were collected.

ABP was obtained through arterial lines connected to pressure transducers measured at the Tragus (Baxter Healthcare Corp. CardioVascular Group, Irvine, CA). Intracranial pressure (ICP) was acquired via an intra-parenchymal strain gauge probe (Codman ICP MicroSensor; Codman & Shurtlef Inc., Raynham, MA), placed in the frontal lobe. Note no EVDs were used to record ICP in this particular study cohort. rSO_2_ was determined with NIRS regional oximetry of the left and right frontal lobes (Covidien INVOS 5100C or 7100). Finally, BIS (an entropy index derived from EEG signals) was recorded bilaterally using the Covidien BIS Complete 4-Channel Monitor (Medtronic of Canada Ltd., Brampton, ON, Canada, www.metronic.com/covidien).

All signals were recorded using digital data transfer or digitized via an A/D converter (DT9803/DT9804/DT9826; Data Translation, Marlboro, MA) and where appropriate sampled at a frequency of 100 Hz, using the ICM+ software (Cambridge Enterprise Ltd., Cambridge, UK, http://icmplus.neurosurg.cam.ac.uk). Signal artifacts were removed using both manual and automated methods prior to further processing or analysis, identical to past work by our lab [13, 14, 39, 40].

Note, NIRS, BIS, and BISopt values were obtained from the hemisphere that had no frontal lobe contusion, overlying hematoma, or subgaleal/scalp hematoma, with visual inspection of the electromyography (EMG) signal of the frontalis indicating no large firing potentials, ensuring no muscle artifacts were present. Finally, no patients had neuromuscular blockade agents administered during the periods of recorded physiology, which may impact BIS values.

### Signal analysis

Signal analysis work was conducted using ICM+ software. Initially all signals were down-sampled using a 10-s non-overlapping moving average filter, in order to focus on the frequency range associated with cerebrovascular reactivity [41, 42]. In so doing rSO_2_ (right and left), BIS (right and left), ICP, and MAP (from ABP) were all produced. COx_a was found over a 5-min moving window between the Pearson’s correlation of rSO_2_ and MAP, output in a minute-by-minute update frequency [25]. PRx was derived using the standard Pearson correlation between 30 consecutive 10-s windows of ICP and MAP, updated every minute [1, 3, 4, 43, 44].

### Optimal depth of sedation determination (BISopt)

To determine BISopt in individual patients, a 60-s median BIS time trend was calculated alongside COx_a. Similar to past work by Aries et al. in optimal cerebral perfusion pressure (CPPopt) determination [6] and our group for BISopt [14], a custom created automatic quadratic curve-fitting method was applied to the binned BIS data to determine the BIS value with the lowest associated COx_a values (for details see Additional file 1A). Notably COx_a index ranges from − 1 to 1, with values higher than ~ 0.3 au (arbitrary units) being linked with impaired cerebrovascular reactivity, thus higher COx_a values are considered to worsen autoregulatory status. With the COx_a values binned into BIS ranges of 4 au, we expect to see some form of U-shaped curve. In this binned plot, from the lowest binned COx_a value its associated BIS value is chosen as BISopt. Using this method, a continuous time trend of BISopt could be calculated, generated from a 4-h moving time window updated every minute. The BISopt curve could be generated when at least 50% of the required data points of COx_a were available, i.e., after a minimum of 2 h of monitoring, in keeping with the standard time window of CPPopt [6–8, 45, 46]. Similarly BISopt was derived using PRx, to facilitate comparison with BISopt derived from COx_a [14].

### Statistical analysis

Statistical analysis was conducted using R software. Descriptive analyses were of BISopt/BIS and its associations with other physiologic parameters of interest in moderate/severe TBI care. All physiologic variables were found to be non-parametric in nature, via Shapiro–Wilks’s testing. Alpha was set at 0.05 with no corrections for multiple comparisons given the exploratory nature of this study.

BIS and COx_a were compared through linear regression to identify their basic relationship. Then the COx_a data were binned into 4 au of BIS for various patients to demonstrate if there existed some form of U-shaped relationship. Additional file 2B gives some clear examples for multiple patients.

Next BISopt was visually obtained from the entire recording period, for each patient, which was indicated by the lowest binned COx_a value. This visually determined BISopt value was compared to the derived quadratic curve-fitting algorithm (Additional file 1A) using a histogram and Wilcoxon signed-ranked test to identify the accuracy between these methods.

Next, the percentage of time that BISopt could be determined for each patient over a 4-h sliding window for the entire recording period, using the previously described algorithm, was determined. Using this % yield of BISopt, we also performed a subgroup analysis by finding the % yield of BISopt and its association with sedative agents/dose (fentanyl, propofol, and ketamine) and vasopressor agents (norepinephrine, phenylephrine, vasopressin, and milrinone). The data were separated for times which BISopt could be found (i.e., where both BIS and COx_a data were available), and the sedative agents/dose over this time were added from our prospectively maintained data obtained from bedside nursing charts. Due to the variability in dose and its weak association with sedation depth, 3 categories of sedation dose were chosen for each agent (high, moderate, and low) [47, 48].

Finally, in the patients who had sufficient data as to determine BISopt with PRx and COx_a, we compared the resulting visually calculated BISopt values using a Wilcoxon signed-ranked test (Additional file 6F).

## Results

### Patient characteristics

**Fig. 1:**
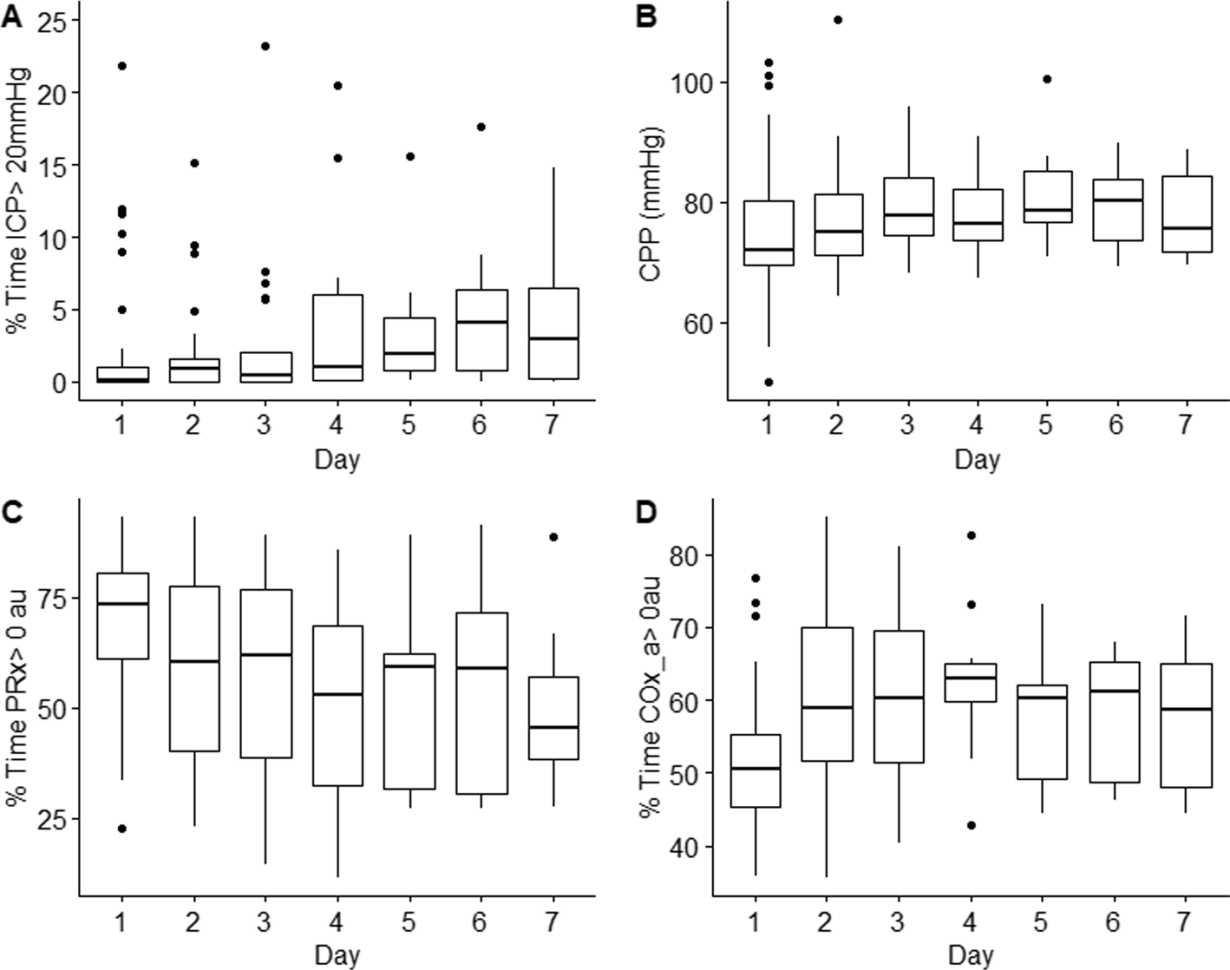
Daily measured cerebral physiology. Demonstrates key daily physiological relationships. **A** Is the % time ICP was above 20 mmHg per day, **B** the median CPP value per day, **C** and **D** were PRx and COx_a > 0 au which is associated with impaired cerebrovascular reactivity. Au, arbitrary units; COx_a, cerebral oximetry index; CPP, cerebral perfusion pressure; ICP, intracranial pressure; mmHg, millimeters of mercury; PRx, pressure reactivity

42 patients were identified with high-frequency NIRS, BIS, and ABP signals present. The median age was 41.5 (interquartile range [IQR]: 24.25–58.5) with 86% being male (37 patients). All patients were on both propofol and/or fentanyl as continuous infusions during the course of the physiologic recordings (Table 1, Fig. 1). The median total duration of paired BIS/NIRS/ABP recording was 33 h (IQR: 13 to 59 h). For impact of infusion details, refer to Additional file 5E.

**Table 1:** 42 patient demographics

Demographics	Median (interquartile range) or number of patients
Age	41.5 (24.25–58.5)
Sex (% male)	37 (86%)
Best admission GCS—total	6.5 (4–8)
Best admission GCS—motor	4 (2–5)
Number with hypoxia episode	11
Number with hypotension episode	7
Number with traumatic SAH	40
Number with epidural hematoma	5
Pupils	
Bilateral unreactive	4
Unilateral unreactive	7
Bilateral reactive	31
Admission Marshall CT	
V	22
IV	6
III	11
II	3
Mean ICP (mmHg)	7.9 (4.2–10.7)
% time ICP > 20 mmHg	0.3 (0.0–2.0)
% time ICP > 22 mmHg	0.1 (0.0–1.2)
Mean CPP (mmHg)	76 (71.3–83.5)
% time CPP > 70 mmHg	74 (52–84)
% time CPP < 60 mmHg	4 (0.4–6.3)
Mean rSO_2_ (au)	68.5 (61.3–76.5)
Mean BIS (au)	45.1 (34.6–60.5)
Mean PRx (au)	0.16 (-0.05–0.27)
% time PRx > 0	68 (55–81)
% time PRx > 0.25	42 (28–57)
% time PRx > 0.35	31 (20–45)
Mean COx_a (au)	0.06 (0.01–0.13)
% time COx_a > 0	54 (49–61)
% time COx_a > 0.25	22 (16–27)

Au, arbitrary units; BIS, bispectral index; COx_a, cerebral oximetry index; CPP, cerebral perfusion pressure; CT, computerized tomography; GCS, Glasgow Coma Score; ICP, intracranial pressure; mmHg, millimeters of mercury; PRx, pressure reactivity; rSO_2_, regional cerebral oxygen saturation; SAH, subarachnoid hemorrhage

### Relationship between BIS and cerebrovascular physiology

**Fig. 2:**
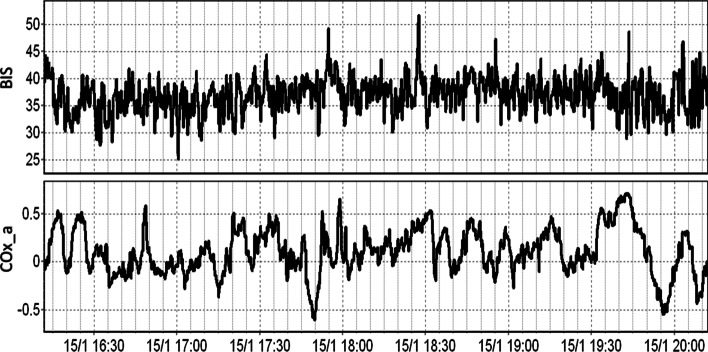
The temporal relationship between BIS and COx_a. The figure shows the BIS and COx_a values over the 4-h period. BIS, bispectral index; COx_a, cerebral oximetry index

**Fig. 3:**
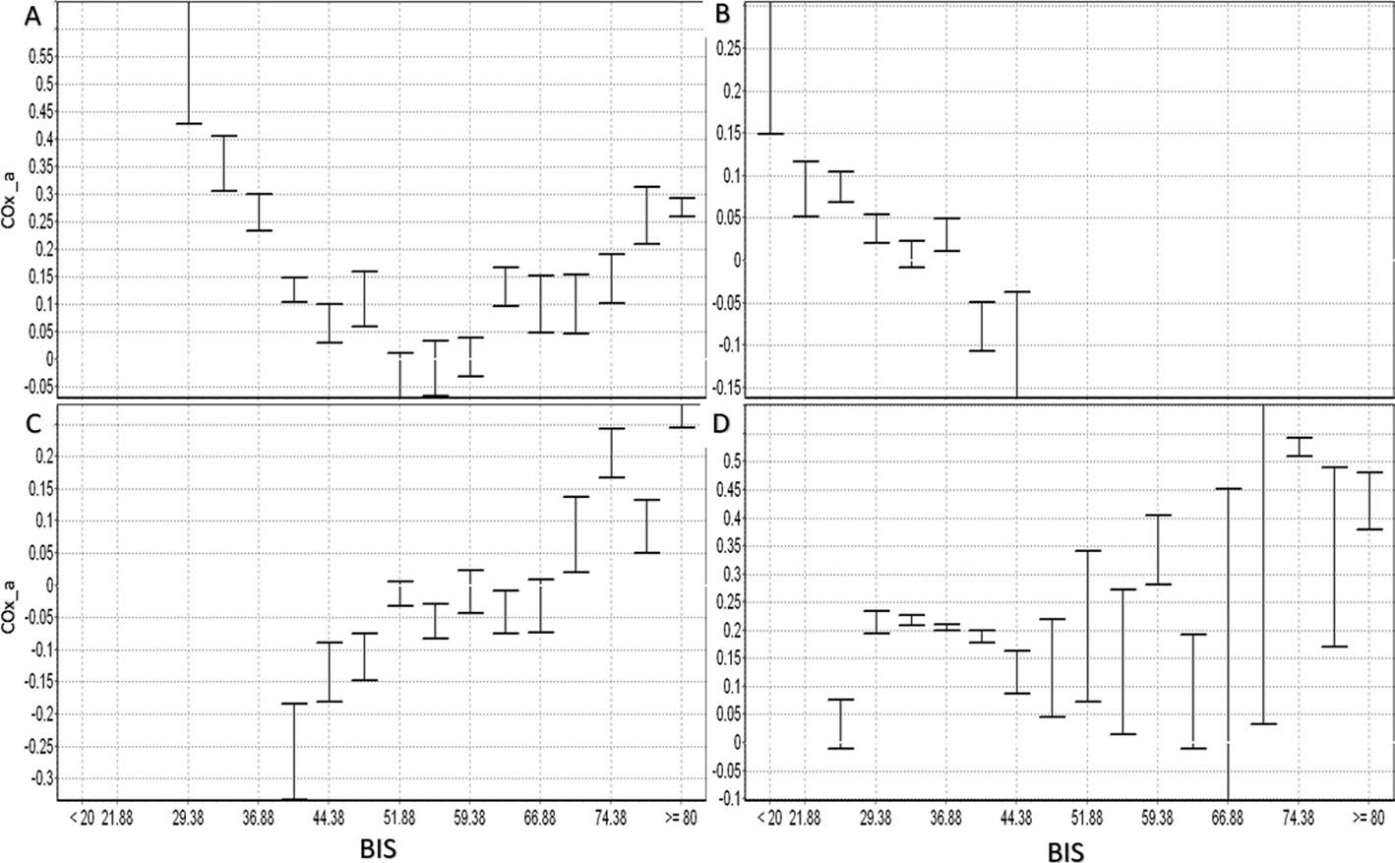
COx_a vs BIS error plots for different patients. Some patient examples of BIS vs COx_a, demonstrating various examples of this relationship. **A** Shows the U-shaped curve on which a full BISopt curve is found. **B** Shows a descending BISopt curve, **C** shows an ascending BISopt curve. **D** Shows an unclear result where BISopt cannot be determined. BIS, bispectral index; COx_a, cerebral oximetry index

The temporal relationship of BIS and COx_a over a 4-h period can be seen for a patient in Fig. 2. Note the high temporal fluctuations in both the BIS and COx_a signals in this example. Similar relationships were seen in all patients. In Fig. 3, error bar plots of COx_a vs BIS for different patients are presented, highlighting the parabolic relationship between COx_a and BIS, similar to that seen between PRx and BIS [13, 14]. In most cases, both light and heavy sedation levels appear to worsen COx_a response (higher values). Though this U-shaped relationship was not always seen, it is in keeping with past results in CPPopt and BISopt studies [6, 13, 14, 24]. There are cases when the relationship curve does not include the convex point, thus the estimated “optimal” value will be either overestimated (ascending curve) or underestimated (descending curve) depending on the shape of the fitted part and therefor it will not have the desired U-shaped curve.

**Fig. 4:**
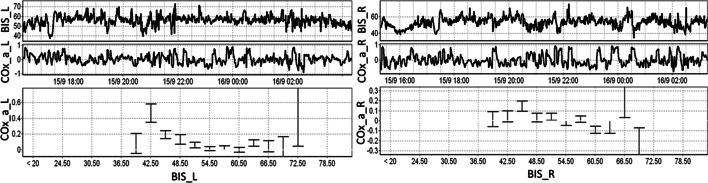
Regional example of COx_a vs BIS. The figure demonstrates the left and right-side example of the BIS and COx_a relationship. BIS_L, left bispectral index; BIS_R, right bispectral index; COx_a_L, left cerebral oximetry index; COx_a_R, right cerebral oximetry index

Figure 4 shows the responses of the left and right BIS and COx_a in the same patient. Though the values and responses vary slightly, for the most part they are similar. More optimal BIS values appearing can be more apparent on one side, thus demonstrating the importance of bilateral monitoring. Full examples of the curves for more patients are in Additional file 2B.

### Relationship between BIS and COx_a—BISopt for entire recording period

In Additional file 2B example plots of BIS vs COx_a for 33 patients that had sufficient data were found (spanning BIS 12 au and COx_a change > 0.1). Additional file 3C is an example of a patient whose COx_a variation failed to meet the 0.1 threshold. As previously stated, there are cases when the BIS vs COx_a curve does not include the convex point, thus the estimated “optimal” value will be either overestimated (ascending curve), underestimated (descending curve) or fail to have an BISopt.

**Fig. 5:**
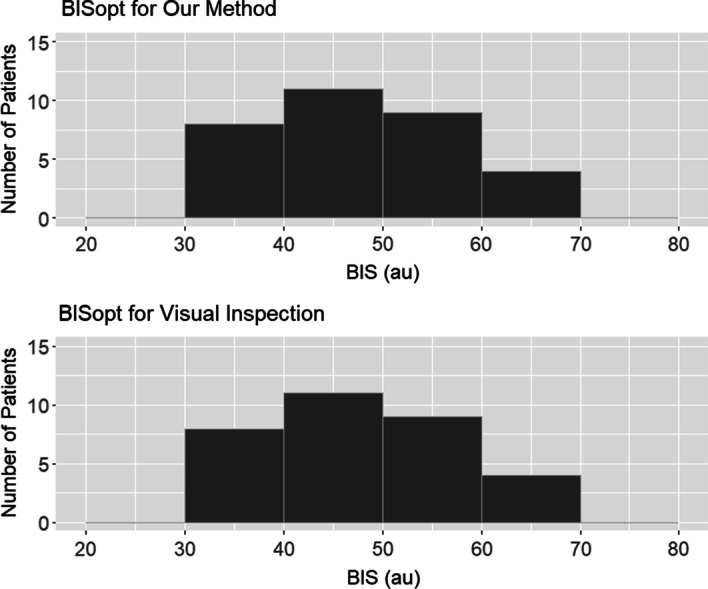
BISopt through our method and visual inspection (33 patients). The figure shows the different BIS values associated with each BISopt method. The Wilcoxon signed-ranked test *p*-value between these two histograms was 0.734, demonstrating that the median values are similar. au, arbitrary units; BIS, bispectral index; BISopt, optimal BIS value

Figure 5 shows histogram plots for the distribution of the BISopt value found using the described algorithm in Additional file 1A and visual inspection for each patient’s full recording time. The Wilcoxon signed-ranked test between the visually and algorithmically determined BISopt was *p* = 0.734, median values of 52 (IQR: 44–56) vs 52 (IQR: 44–56), note that the number of patients was smaller than the total as some values of BISopt could not be determined over the full data of a patient due to the limited COx_a variation.

### Relationship between BIS and COx_a over 4-h windows—continuously updating BISopt

Like continuous measures of PRx derivation, the variation of COx_a and BISopt would need to be optimally assessed and updated continuously. The continuous optimal COx_a relationship was highlighted in a patient with uninterrupted BIS and COx_a data, seen in Additional file 4D. The data were separated in 4-h window lengths and used to generate an error bar plot of the relationship.

Additional file 5E1 and E2 identifies the % yield and its association between sedative and vasopressor agents. This highlights the fact that BISopt appears to be calculable regardless of the patient’s agent state, with similar results seen in BISopt calculated through PRx [14]. The overall % yield for each patient is 47% (IQR: 31–62), this yield % was similar and independent of sedative or vasopressor agent. Thus the feasibility of generating an optimal, or partial optimal, curve is in keeping with previously published CPPopt and BISopt methodologies, with similar % yields [6, 13, 24].

### Relationship between BISopt COx_a vs PRx

Within Additional file 6F, a comparison of the BISopt derived through COx_a vs PRx was indicated. For all patients the determination of PRx, BIS and COx_a was possible, however the ability to derive BISopt from both PRx and COx_a was limited in some patients. This is due to a variety of factors including a limited time of good monitoring of NIRS/BIS and limited variance in BIS/COx_a/PRx, which limits our ability to derive BISopt.

Though there were some variations between the derived BISopt, median values of 48 (IQR: 40–56) vs 45 (IQR: 40–56) for COx_a vs PRx BISopt, there was no statistically significant difference (Wilcoxon signed-ranked test *p*-value = 0.31). Furthermore, in cases where both PRx and COx_a had clear U-shaped curves, the resulting BISopt values were mostly identical (varying only at most one bin away).

## Discussion

In this study, we examined the relationship between cerebrovascular reactivity and depth of sedation as determined via COx_a and BIS. To our knowledge, this is the first report of a potential method for non-invasively determining an optimal depth of sedation, BISopt, with respect to cerebrovascular reactivity; opening the door to applications beyond TBI patients. Furthermore, in line with past work within CPPopt and BISopt (based on PRx), similar descriptive relationships were found between COx_a and BIS [6, 13, 14, 24]. Secondly, similar to previous studies using PRx, there appears to be an optimal sedation depth as determined by a “U-shaped” relationship between COx_a and BIS. This COx_a-based BISopt was found to be calculable in most patients despite various sedative or vasopressor agents used. Importantly, the values of BISopt derived through COx_a were found to be concordant with values of BISopt as determined invasively through PRx.

While COx_a utility remains in its infancy, there is a growing literature body that evaluates the similarity between NIRS-based cerebrovascular reactivity measures and the more standard PRx [25–30]. This is evidenced by pre-clinical literature providing only validation for ICP and NIRS-based cerebrovascular reactivity indices as true measures of the lower limit of autoregulation in several different animal models [31]. This strong association between NIRS and cerebral blood flow/cerebral blood volume is also supported by the growing objective body of literature supporting a moderate/strong correlation between NIRS measures and cerebral blood flow [49, 50]. Similarly, time-series and multi-variate clustering analysis has demonstrated strong co-variance between NIRS-based measures and standard PRx [26, 28, 29]. Thus, we feel that comparing COx_a and PRx is in keeping with the growing established literature body on NIRS-based cerebrovascular reactivity in TBI. Our findings that BISopt was similar between COx_a and PRx, is in keeping with the prior literature supporting similarities between NIRS and ICP-derived cerebrovascular reactivity metrics.

The parabolic relationship between BIS sedation depth and COx_a may offer a unique method of determining and targeting a so-called optimal depth of sedation (BISopt). The robustness of this non-invasive BISopt is demonstrated by the fact that this parabolic relationship between COx_a and BIS levels was present in many patients in this cohort over their entire ICU stay. The interpretation of this persistent relationship is that both light and heavy sedation depths, in moderate/severe TBI, may result in derangements to cerebrovascular autoregulation [25, 51].

Sedation depth has previously been known to impact cerebral metabolic state, with deeper sedation depths resulting in reduced metabolic requirements and as such reduced cerebral blood flow [13, 16, 52]. In a metabolically suppressed state the cerebrovascular response is blunted and thus cerebral autoregulation becomes impaired [53, 54]. Therefore, we speculate the existence of an optimal sedation value which achieves a balance between metabolic demand and control. However, there are still many limitations with this interpretation as BIS/NIRS monitoring has its own limitations in measuring the desired physiology as well as some patients having no notable BISopt.

As there are many patient characteristics that impact the determination of BISopt, there were patients in this cohort where BISopt could not be determined over the full time. This is due to a variety of factors including a limited time of good monitoring of NIRS/BIS, limited variance in BIS/COx_a/PRx, and algorithm limitations, thus this limits our ability to derive BISopt in an individual patient.

Additionally, the impact of various physiological factors on these derived measures is still under-explored. None of the patients included in this study had clinical seizures or were found to be in status epilepticus, which could significantly impact the validity of BIS monitoring as a surrogate for depth of sedation. As well it should be noted that in this population no patients had large frontalis EMG artifacts that are known to impact BIS recordings.

For our data collection, we leveraged spatially resolved NIRS techniques to help remove scalp contamination by utilizing a proprietary spatially resolved methodology where short-channel scalp signal is removed from the long-channel data, to provide a true cerebral signal—reducing the risk of contamination.

This technique does have limitations to its depth of penetration. As such, the impact of extravascular blood on NIRS monitoring was accounted for crudely through the exclusion of data collected over regions where large intra- or extra-axial hematomas were present. This included cerebral contusions as well as subdural, epidural, and subgaleal hematomas. Obviously, this limits the generalizability of this study, especially in the TBI population. Interestingly, data from such regions generally yields, somewhat expectedly, minimal variability in NIRS signals over the frequency ranges evaluated by COx_a. The corollary of this is that COx_a values from such regions are constant at 0 and independent of changes in depth of sedation, thereby being prone to a type 2 error in the identification of BISopt, as opposed to a type 1 error. All of this highlights the need for further evaluation in neurotrauma populations, involving an analysis of a variety patient states known to impact BIS and NIRS monitoring.

Next, there was some hemispherical similarities in patients without hemispheric contusions and good physiological recordings/variance of the right and left BIS and COx_a. When the method was working at its best, with a clear U-shaped curve and high variance in COx_a/BIS there was a near perfect agreement with the measures. Moreover, the discrepancy of the BISopt value of 5 or even 10 au is reasonable as there is a limitation with the device as well as the ability to target such a specific sedation index (BIS is not vary precise).

However, the extent to which this occurs in all patient populations needs to be further verified, with patient cerebral hemisphere state (contusion, hematoma and impaired cerebral reactivity) clearly known to interfere with BIS and COx_a determination. Furthermore, the natural limitations with the devices as well as the clinical experience of the patient both impact how well BISopt can be determined. Likewise hemispheric asymmetry in cerebrovascular reactivity has been seen in past TBI and other neurocritical ill populations that use primarily transcranial Doppler to continuously assess cerebral autoregulation [55–57]. Finally, asymmetry in cortical electrophysiology (and thus entropy estimations) could also account for hemispheric differences in the derived BISopt. Thus, before BISopt can be fully implemented, the discrepancy presented with regional differences needs to be understood.

Cerebral autoregulation monitoring is an expanding concept, with growing interest in fields outside of TBI. Literature within cardiac arrest patients has shown a narrowing of intact cerebral autoregulation states [58]. Likewise, in patients with subarachnoid hemorrhage, cerebral autoregulation has been shown to become impaired, which has been implicated with the development of delayed cerebral ischemia assessed through various modalities including NIRS methods [59, 60]. After a stroke, cerebral autoregulation has shown to be impaired with transcranial Doppler indices [61]. As BISopt can be determined non-invasively, it could be adapted to these pathological states, though considerably more investigation is required.

Finally, the automated algorithm for continuously determining BISopt has also been described in this study and was shown to emulate BISopt as determined manually by visual inspection. Despite its non-invasive nature, this method has a resulting yield similar to previously outlined methods for calculating BISopt which relied on the invasively derived PRx [6, 7, 13, 14, 24, 62]. Ultimately, this algorithm may allow for the continuous generation of a non-invasively derived BISopt in real-time at the bedside.

## Limitations

This study is preliminary and exploratory in nature, with TBI populations having notable heterogeneity, thus future exploration is required. Though currently we have not been able to identify a particular pattern of an individual more conducive to BISopt calculations, there are many foreseeable pathophysiological factors that could impact BISopt. Type of injury, patient genetic profiles known to impact TBI outcome and different sedation/vasopressor regimes could all play a role in the ability to assess and derive these methods and will need to be assessed in further studies. Second, though BIS and NIRS are established, the effectiveness of such methods requires further exploration within TBI and other cohorts. It is well known that other physiological factors outside brain vasculature can impact NIRS response and thus this will need to be addressed before this method can be adopted.

In this analysis we believe that there are several reasons for lack of BISopt derivation including: time of data recorded, variance in both BIS/NIRS and a limitation in the algorithm. As both BIS/NIRS require direct skin contact on the forehead, in the application of these devices we found that they fall off or lose contact to the patient resulting in a loss of signal recorded. Additionally, it is always more advantageous to assess BISopt in patients who have large variations in BIS/NIRS over time of care. As this was an observational study, some patients were more stable throughout recording resulting in no significant changes to BIS/NIRS. These issues need to be addressed if BISopt derivation is to improve.

In our preliminary results, BISopt could be calculated in patients with various sedative agents/amounts, however previous literature has noted that physiological states are influenced by administered anesthesia [48, 63, 64]. Metabolic suppression would never be conducive to the BIS device, with optimal BIS ranges between 20–80 au, though within these bounds the best sedation depth is still unknown. Thus, to what extent sedative agent/amount impacts BISopt is still undetermined.

To calculate BISopt, it is not the impairment of autoregulation alone that is important but the impairment of autoregulation and variance in BIS together which allows for the full assessment of sedation depth on cerebral autoregulation. Again, as this is an observational study, we are limited by the changes in BIS over the patient care and this is a big limitation in BISopt derivation.

Finally, the current BISopt algorithm has many of its own limitations, including: low percent yield, high variation in certain patients and feasibility. Moreover, the optimal window of time to calculate and update both BISopt and COx_a is limited. As well, BISopt’s association with outcome including detailed neuro-cognitive and functional outcome done serially over the long term is needed to fully highlight this methods value and impact. Likewise, the way in which rSO_2_/COx_a are handled pre-BISopt determination may play a role in the resulting BISopt index, thus further exploration into these methods needs to be performed. Furthermore, BIS as a measure of sedation requires further validation.

The feasibility of BISopt and to a lesser extent COx_a is still being determined. The entire set of physiological factors influencing these parameters is not fully understood and the optimal methods of computing these parameters has not been determined. Moreover, BISopt as a methodology is currently limited by the variance in both BIS and NIRS of the patient. In order for this method to work effectively their needs to be variance in these measures to document the upper/lower limits of sedation vs cerebral autoregulation.

Such a personalized BISopt target needs to be explored in both TBI and non-TBI populations, in association with long-term neuro-cognitive and functional outcome measures that are sampled serially. It remains uncertain if BISopt targeting reduces morbidity related to sedation and if it is applicable outside of TBI.

## Future directions

Future work in this area would benefit from longer BIS and NIRS recordings, alongside the comparison of other cerebrovascular reactivity methods, allowing for sliding window analysis similar to the continuously updating CPPopt calculations. Based on these promising preliminary findings, we currently have an ongoing prospective observational study at our center. We will continue to record the relationship between BIS-defined sedation depth and multi-modal monitoring assessed cerebral physiology in moderate/severe TBI, in order to expand our dataset substantially over the next 2 years. With future work in this area, we hope to further define the relationship between sedation administration/depth of sedation and cerebrovascular response in TBI. Within our ongoing data collection and assessment, we will expand NIRS/BIS methods into non-TBI patients, notably patients undergoing elective spine surgeries. As this is one of the only labs in the world that connects continuous physiology to high-frequency therapeutic information, we will be able to better comment on the various patient characteristics that may impact BISopt calculation including sedation depth/agent. Likewise, with the expansion of this dataset, we will be able to better comment on outliers and potential co-variant factors as well as the differences in hemispherical asymmetry.

We are currently assessing different methods and techniques to calculate/assess cerebral autoregulation response, like those in a spectral domain. Such methods can be easily applied to the BISopt calculation which may improve the limitations in calculation and yields. Moreover, we are exploring methods to improve percent yield for the CPPopt index, which we would hope to have similar improvements to BISopt yields, given their similarities.

Furthermore, the BIS sedation depth index was chosen as in Canada it is one of the only commercially and readily available products to measure sedation depth in the intensive care unit. However, there are other methods to calculate sedation depth, with entropy commonly used to find depth of sedation. Future research into the BISopt concept would benefit from exploration of different measures of entropy including: multiscale, sample, approximation and weighted.

## Conclusion

This exploratory study highlights the potential impact of depth of sedation on non-invasive determination of cerebrovascular reactivity in TBI. This work expands on past literature which suggests that there may be an individual optimal depth of sedation, so as to optimize cerebrovascular reactivity. However, further study on depth of sedation and its impact on cerebrovascular physiology is required, as there exists many under-explored factors with this method. With further validation, the BISopt concept using the above-characterized non-invasive method carries the potential to facilitate personalized depth of sedation targeting for the larger general ICU population requiring sedation and mechanical ventilation, however more preliminary studies on the other co-factors must be performed.

## Supplementary Information

**Additional file 1.** **Supplementary File A.** Method to Determine the Optimal BIS Value.

**Additional file 2.** **Supplementary Figures B.** Examples of 33 Patient’s BISopt Derivation – Entire Recording.

**Additional file 3.** **Supplementary Figures C.** Example of Patient with “Too Flat” Plot.

**Additional file 4.** **Supplementary Figures D.** Sequential Error Bar Plotting.

**Additional file 5.** **Supplementary Figures E.** BISopt % Yeild.

**Additional file 6.** **Supplementary Figures F.** COx_a vs PRx BISopt Values (33 Patients).

## Data Availability

All data are presented in this manuscript or the additional materials.

## Abbreviations

ABP: Arterial blood pressure
BIS: Bispectral index
BISopt: Optimal bispectral index
COx_a: Cerebral oximetry index
CPP: Cerebral perfusion pressure
CPPopt: Optimal cerebral perfusion pressure
ICP: Intracranial pressure
ICU: Intensive care unit
NIRS: Near-infrared spectroscopy
PRx: Pressure reactivity index
rSO_2_: Regional brain tissue oxygen saturation
TBI: Traumatic brain injury

## References

[1] Donnelly J, Czosnyka M, Adams H (2019). Twenty-five years of intracranial pressure monitoring after severe traumatic brain injury: a retrospective, single-center analysis. Neurosurgery.

[2] Bennis FC, Teeuwen B, Zeiler FA (2020). Improving prediction of favourable outcome after 6 months in patients with severe traumatic brain injury using physiological cerebral parameters in a multivariable logistic regression model. Neurocrit Care.

[3] Czosnyka M, Smielewski P, Kirkpatrick P (1997). Continuous assessment of the cerebral vasomotor reactivity in head injury. Neurosurgery.

[4] Sorrentino E, Diedler J, Kasprowicz M (2012). Critical thresholds for cerebrovascular reactivity after traumatic brain injury. Neurocrit Care.

[5] Zeiler FA, Ercole A, Beqiri E (2019). Association between cerebrovascular reactivity monitoring and mortality is preserved when adjusting for baseline admission characteristics in adult traumatic brain injury: a CENTER-TBI study. J Neurotrauma.

[6] Aries MJ, Czosnyka M, Budohoski K (2012). Continuous determination of optimal cerebral perfusion pressure in traumatic brain injury*. Crit Care Med.

[7] Steiner L, Czosnyka M, Piechnik S (2002). Continuous monitoring of cerebrovascular pressure reactivity allows determination of optimal cerebral perfusion pressure in patients with traumatic brain injury. Crit Care Med.

[8] Kramer AH, Couillard PL, Zygun DA (2019). Continuous assessment of “optimal” cerebral perfusion pressure in traumatic brain injury: a cohort study of feasibility, reliability, and relation to outcome. Neurocrit Care.

[9] Carney N, Totten AM, O’Reilly C (2017). Guidelines for the management of severe traumatic brain injury, fourth edition. Neurosurgery.

[10] Adams H, Donnelly J, Czosnyka M (2017). Temporal profile of intracranial pressure and cerebrovascular reactivity in severe traumatic brain injury and association with fatal outcome: an observational study. PLoS Med.

[11] Zeiler FA, Beqiri E, Cabeleira M (2020). Brain tissue oxygen and cerebrovascular reactivity in traumatic brain injury: a collaborative European NeuroTrauma Effectiveness Research in Traumatic Brain Injury Exploratory Analysis of Insult Burden. J Neurotrauma.

[12] Zeiler FA, Ercole A, Beqiri E (2019). Cerebrovascular reactivity is not associated with therapeutic intensity in adult traumatic brain injury: a CENTER-TBI analysis. Acta Neurochir.

[13] Froese L, Dian J, Gomez A, Zeiler FA (2021). Sedation and cerebrovascular reactivity in traumatic brain injury: another potential for personalized approaches in neurocritical care?. Acta Neurochir.

[14] Froese L, Gomez A, Sainbhi AS (2022). Continuous determination of the optimal bispectral index value based on cerebrovascular reactivity in moderate/severe traumatic brain injury: a retrospective observational cohort study of a novel individualized sedation target. Crit Care Explor.

[15] Kochanek PM, Tasker RC, Bell MJ (2019). Management of pediatric severe traumatic brain injury: 2019 consensus and guidelines-based algorithm for first and second tier therapies. Pediatr Crit Care Med.

[16] Froese L, Dian J, Batson C (2020). cerebrovascular response to propofol, fentanyl, and midazolam in moderate/severe traumatic brain injury: a scoping systematic review of the human and animal literature. Neurotrauma Rep.

[17] Zeiler FA, Sader N, Gillman LM (2016). The cerebrovascular response to ketamine: a systematic review of the animal and human literature. J Neurosurg Anesthesiol.

[18] Flower O, Hellings S (2012). Sedation in traumatic brain injury. Emerg Med Int.

[19] Girard TD (2018). Sedation, delirium, and cognitive function after critical illness. Crit Care Clin.

[20] Porhomayon J, El-Solh AA, Adlparvar G (2016). Impact of sedation on cognitive function in mechanically ventilated patients. Lung.

[21] Stephens RJ, Dettmer MR, Roberts BW (2018). Practice patterns and outcomes associated with early sedation depth in mechanically ventilated patients: a systematic review and meta-analysis. Crit Care Med.

[22] Budohoski KP, Czosnyka M, de Riva N (2012). The relationship between cerebral blood flow autoregulation and cerebrovascular pressure reactivity after traumatic brain injury. Neurosurgery.

[23] Thelin EP, Raj R, Bellander B-M (2019). Comparison of high versus low frequency cerebral physiology for cerebrovascular reactivity assessment in traumatic brain injury: a multi-center pilot study. J Clin Monit Comput.

[24] Beqiri E, Smielewski P, Robba C (2019). Feasibility of individualised severe traumatic brain injury management using an automated assessment of optimal cerebral perfusion pressure: the COGiTATE phase II study protocol. BMJ Open.

[25] Brady KM, Lee JK, Kibler KK (2007). Continuous time-domain analysis of cerebrovascular autoregulation using near-infrared spectroscopy. Stroke.

[26] Zeiler FA, Donnelly J, Menon DK (2017). Continuous autoregulatory indices derived from multi-modal monitoring: each one is not like the other. J Neurotrauma.

[27] Zeiler FA, Donnelly J, Calviello L (2017). Pressure autoregulation measurement techniques in adult traumatic brain injury, part I: a scoping review of intermittent/semi-intermittent methods. J Neurotrauma.

[28] Zweifel C, Castellani G, Czosnyka M (2010). Noninvasive monitoring of cerebrovascular reactivity with near infrared spectroscopy in head-injured patients. J Neurotrauma.

[29] Mathieu F, Khellaf A, Ku JC (2020). Continuous near-infrared spectroscopy monitoring in adult traumatic brain injury: a systematic review. J Neurosurg Anesthesiol.

[30] Gomez A, Sainbhi AS, Froese L (2021). Near infrared spectroscopy for high-temporal resolution cerebral physiome characterization in TBI: a narrative review of techniques, applications, and future directions. Front Pharmacol.

[31] Sainbhi AS, Froese L, Gomez A (2021). Continuous time-domain cerebrovascular reactivity metrics and discriminate capacity for the upper and lower limits of autoregulation: a scoping review of the animal literature. Neurotrauma Rep.

[32] Liu X, Akiyoshi K, Nakano M (2021). Determining thresholds for three indices of autoregulation to identify the lower limit of autoregulation during cardiac surgery. Crit Care Med.

[33] Lee JK, Yang Z-J, Wang B (2012). Noninvasive autoregulation monitoring in a swine model of pediatric cardiac arrest. Anesth Analg.

[34] Brady KM, Mytar JO, Kibler KK (2010). Noninvasive autoregulation monitoring with and without intracranial pressure in the Naïve Piglet Brain. Anesth Analg.

[35] Lee JK, Kibler KK, Benni PB (2009). Cerebrovascular reactivity measured by near-infrared spectroscopy. Stroke.

[36] Brady KM, Lee JK, Kibler KK (2008). Continuous measurement of autoregulation by spontaneous fluctuations in cerebral perfusion pressure. Stroke.

[37] Chesnut R, Aguilera S, Buki A (2020). A management algorithm for adult patients with both brain oxygen and intracranial pressure monitoring: the Seattle International Severe Traumatic Brain Injury Consensus Conference (SIBICC). Intensive Care Med.

[38] Scheeren TWL, Schober P, Schwarte LA (2012). Monitoring tissue oxygenation by near infrared spectroscopy (NIRS): background and current applications. J Clin Monit Comput.

[39] Froese L, Dian J, Batson C (2020). The impact of hypertonic saline on cerebrovascular reactivity and compensatory reserve in traumatic brain injury: an exploratory analysis. Acta Neurochir (Wien).

[40] Froese L, Dian J, Batson C (2020). The impact of vasopressor and sedative agents on cerebrovascular reactivity and compensatory reserve in traumatic brain injury: an exploratory analysis. Neurotrauma Rep.

[41] Howells T, Johnson U, McKelvey T, Enblad P (2015). An optimal frequency range for assessing the pressure reactivity index in patients with traumatic brain injury. J Clin Monit Comput.

[42] Fraser CD, Brady KM, Rhee CJ (2013). The frequency response of cerebral autoregulation. J Appl Physiol.

[43] Zeiler FA, Lee JK, Smielewski P (2018). Validation of intracranial pressure-derived cerebrovascular reactivity indices against the lower limit of autoregulation, part II: experimental model of arterial hypotension. J Neurotrauma.

[44] Depreitere B, Citerio G, Smith M (2021). Cerebrovascular autoregulation monitoring in the management of adult severe traumatic brain injury: a delphi consensus of clinicians. Neurocrit Care.

[45] Depreitere B, Güiza F, Van den Berghe G (2014). Pressure autoregulation monitoring and cerebral perfusion pressure target recommendation in patients with severe traumatic brain injury based on minute-by-minute monitoring data. J Neurosurg.

[46] Donnelly J, Czosnyka M, Adams H (2017). Individualizing thresholds of cerebral perfusion pressure using estimated limits of autoregulation. Crit Care Med.

[47] Haberland CM, Baker S, Liu H (2011). Bispectral index monitoring of sedation depth in pediatric dental patients. Anesth Prog.

[48] Duchateau F-X, Saunier M, Larroque B (2014). Use of bispectral index to monitor the depth of sedation in mechanically ventilated patients in the prehospital setting. Emerg Med J.

[49] Sainbhi AS, Gomez A, Froese L, et al (2022) Non-invasive and minimally-invasive cerebral autoregulation assessment: a narrative review of techniques and implications for clinical research. 10.17863/CAM.84398 10.3389/fneur.2022.872731 PMC9087842 35557627

[50] Gomez A, Sainbhi AS, Froese L (2022). The quantitative associations between near infrared spectroscopic cerebrovascular metrics and cerebral blood flow: a scoping review of the human and animal literature. Front Netw Physiol.

[51] Miller S, Mitra K (2017). NIRS-based cerebrovascular regulation assessment: exercise and cerebrovascular reactivity. Neurophotonics.

[52] Ferrarri F, Kelsall AWR, Rennie JM, Evans DH (1994). The relationship between cerebral blood flow velocity fluctuations and sleep state in normal newborns. Pediatr Res.

[53] Lee JH, Kelly DF, Oertel M (2001). Carbon dioxide reactivity, pressure autoregulation, and metabolic suppression reactivity after head injury: a transcranial Doppler study. J Neurosurg.

[54] Steiner LA, Johnston AJ, Chatfield DA (2003). The effects of large-dose propofol on cerebrovascular pressure autoregulation in head-injured patients. Anesth Analg.

[55] Vavilala MS, Tontisirin N, Udomphorn Y (2008). Hemispheric differences in cerebral autoregulation in children with moderate and severe traumatic brain injury. Neurocrit Care.

[56] Lang EW, Yip K, Griffith J (2003). Hemispheric asymmetry and temporal profiles of cerebral pressure autoregulation in head injury. J Clin Neurosci.

[57] Budohoski KP, Czosnyka M, Kirkpatrick PJ (2015). Bilateral failure of cerebral autoregulation is related to unfavorable outcome after subarachnoid hemorrhage. Neurocrit Care.

[58] van den Brule JMD, van der Hoeven JG, Hoedemaekers CWE (2018). Cerebral perfusion and cerebral autoregulation after cardiac arrest. Biomed Res Int.

[59] Budohoski KP, Czosnyka M, Smielewski P (2013). Cerebral autoregulation after subarachnoid hemorrhage: comparison of three methods. J Cereb Blood Flow Metab.

[60] Budohoski KP, Czosnyka M, Kirkpatrick PJ (2013). Clinical relevance of cerebral autoregulation following subarachnoid haemorrhage. Nat Rev Neurol.

[61] Aries MJH, Elting JW, De Keyser J (2010). Cerebral autoregulation in stroke. Stroke.

[62] CPPopt Trends COGiTATE ». https://cppopt.org/cppopt-trend-icm-relase-8-4-4-4/. Accessed 29 Aug 2021

[63] Lesser GS, Friedman R, Deal E (2004). Use of Bispectral Index Monitor (BIS) to follow depth of sedation in patients undergoing colonoscopy with propofol sedation. Gastrointest Endosc.

[64] Sleigh JW, Andrzejowski J, Steyn-Ross A, Steyn-Ross M (1999). The Bispectral Index: a measure of depth of sleep?. Anesth Analg.

