# Supplementary material for: Optimal bispectral index level of sedation and cerebral oximetry in traumatic brain injury: a non-invasive individualized approach in critical care?
Source: Intensive Care Med Exp. 2022 Aug 13;10:33. doi: 10.1186/s40635-022-00460-9 (PMC9375800; doi:10.1186/s40635-022-00460-9)
Supplement: Supplementary file 1 — Additional file 1. Supplementary File A. Method to Determine the Optimal BIS Value. [file 40635_2022_460_MOESM1_ESM.docx]

**Supplementary File A. Method to Determine the Optimal BIS Value**

The method outlined here is similar to the work done by Aries et al. on the determination of CPPopt and Froese et al. on the determination of BISopt.(1, 2) Initially COx_a data were processed using Fisher Transform to achieve a normal distribution eliminating the ceiling effect of the maximum COx_a value of ±1.(3) These COx_a values were divided and averaged into BIS bins spanning 4 arbitrary units (au), chosen as 20 bins over a BIS range from 20 to 80 au. BIS values can range from 0 (isoelectric EEG) to 100 (awake) (4, 5). BIS values from 0 to 20 are EEG burst suppression and 80 to 100 are semi-conscious states, thus have been excluded from the assessment of BISopt (4, 5). The mean value and SD of each bin were then plotted against the bin mean BIS value in order to create the error bar chart representing the relationship between COx_a and BIS. Theoretically, a U-shaped relationship should be demonstrated with cerebrovascular pressure reactivity getting worse (COx_a increasing) for BIS values further away from the curve center. Thus, a quadratic algorithm was therefore used to fit a parabolic curve to the COx_a-BIS error bar plot in order to estimate an optimal BIS value (BISopt), which is the BIS value for which COx_a achieves the smallest value.

The following summarizes the process implemented:

1. Discard BIS bins that contain <2% of the data points.
2. Ensure that the BIS values span at least 3 bins (12 au.).
3. Ensure that COx_a values span at least 0.1 au.
4. Fit a second-order polynomial to the boxplot, the fitted curve must fulfill the following criteria:
   1. In the first attempt, the curve fitted is expected to include a convex point (a sign change of the first derivative from negative to positive). If such a curve cannot be found or it does not fulfill all the remaining criteria, then the monotonically ascending or descending part of the curve can be used. However, the curve must follow a positive convex shape to some extent.
   2. The sequence of the mean COx_a values of the last two bins at each edge of the curve must follow the correct, expected order depending on the part of the parabolic curve fitted (i.e., descending at the left edge and ascending on the right when fitting a parabolic curve including a clear minimum). The edge bins that do not fulfill this criterion are excluded, and the fitting process is repeated.
   3. Data corresponding to the bins used in successful curve fitting (i.e., after various exclusions mentioned above) must at least:
      1. represent 50% of all the data points in the analyzed window period
      2. cover at least 50% of the range of COx_a data available in that period
      3. represent 12 au. of BIS fluctuation, so the number of bins used for data fit must be at least 3
      4. fitted part of the curve must span the range of COx_a values of at least 0.1; in other words, curves that are too “flat” are rejected

5. If all the criteria/restrictions are fulfilled, the fitting procedure is stopped and BISopt is determined. The BISopt value corresponds to the minimum point of the fitted curve that lies within the range of BIS values. It should be noted that when the fitted curve does not include a clear convex point, the estimated optimal value will be either underestimated (ascending curve) or overestimated (descending curve) depending on the shape of the fitted part.

6. If all attempts have been exhausted and no satisfactory curve was fitted, the procedure returns an invalid value (i.e., Not-A-Number value) for the selected period.

References:

1. Aries MJ, Czosnyka M, Budohoski K, et al.: Continuous determination of optimal cerebral perfusion pressure in traumatic brain injury*. *Critical Care Medicine* 2012; 40:2456–2463

2. Froese L, Gomez A, Sainbhi AS, et al.: Continuous Determination of the Optimal Bispectral Index Value Based on Cerebrovascular Reactivity in Moderate/Severe Traumatic Brain Injury: A Retrospective Observational Cohort Study of a Novel Individualized Sedation Target. *Crit Care Explor* 2022; 4:e0656

3. Czosnyka M, Miller C, Participants in the International Multidisciplinary Consensus Conference on Multimodality Monitoring: Monitoring of cerebral autoregulation. *Neurocrit Care* 2014; 21 Suppl 2:S95-102

4. Mitchell-Hines T, Ellison K, Willis S: Using bispectral index monitoring to gauge depth of sedation/analgesia. *Nursing2020 Critical Care* 2017; 12:12–16

5. Mathur S, Patel J, Goldstein S, et al.: Bispectral Index [Internet]. In: StatPearls. Treasure Island (FL): StatPearls Publishing; 2020. [cited 2020 Nov 7] Available from: http://www.ncbi.nlm.nih.gov/books/NBK539809/
