# Supplementary material for: Optimal bispectral index level of sedation and cerebral oximetry in traumatic brain injury: a non-invasive individualized approach in critical care?
Source: Intensive Care Med Exp. 2022 Aug 13;10:33. doi: 10.1186/s40635-022-00460-9 (PMC9375800; doi:10.1186/s40635-022-00460-9)

**Supplementary Figures B. Examples of 33 Patient’s BISopt Derivation – Entire Recording**

Boxplots represent the right or left side binned BIS values and the Fisher Z COx_a values. Plots have been omitted for a patient’s hemisphere having any factors that interfere with a BIS signal (hemisphere with hematoma and/or contusion) or bins were similar across the whole domain. The line is a curve of our method on the data over the full recording patient data, with the blue dot indicating the BISopt value. For the bins where data is less than 2%, there will be no curve line, thus the curve will not extend to the full data span. au, arbitrary units; BIS, bispectral index; COx_a, Cerebral oximetry index

Patient 1 – Descending Curve


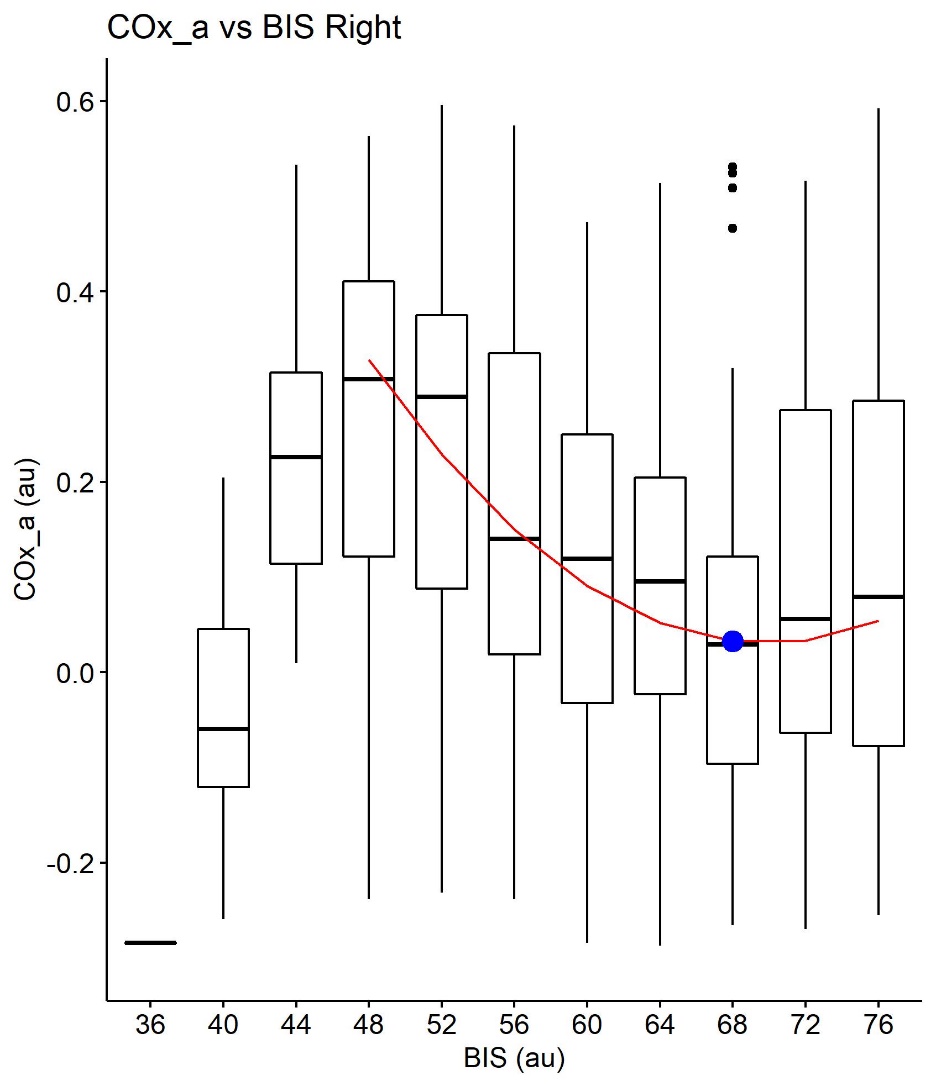


Patient 2 – U-Shaped Curve


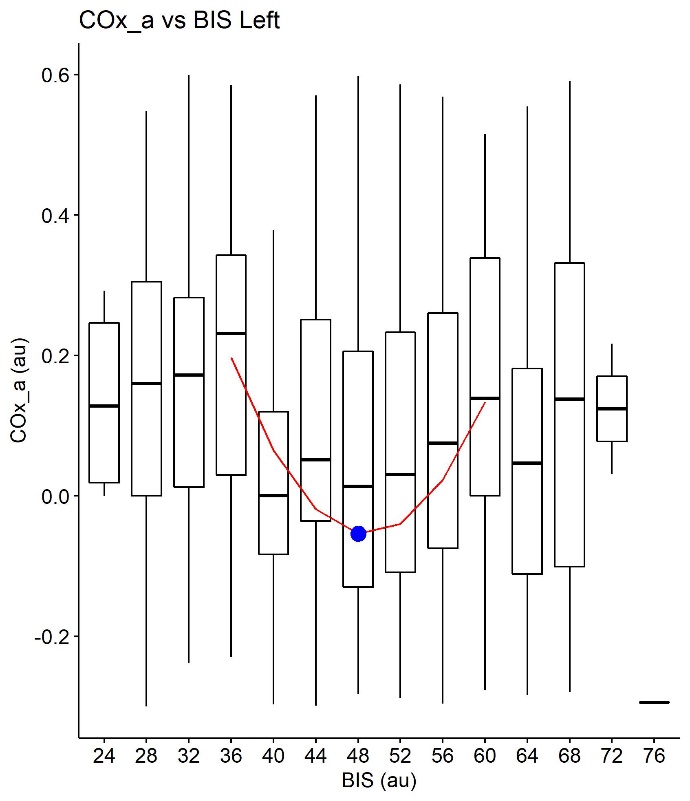


Patient 3– Ascending Curve


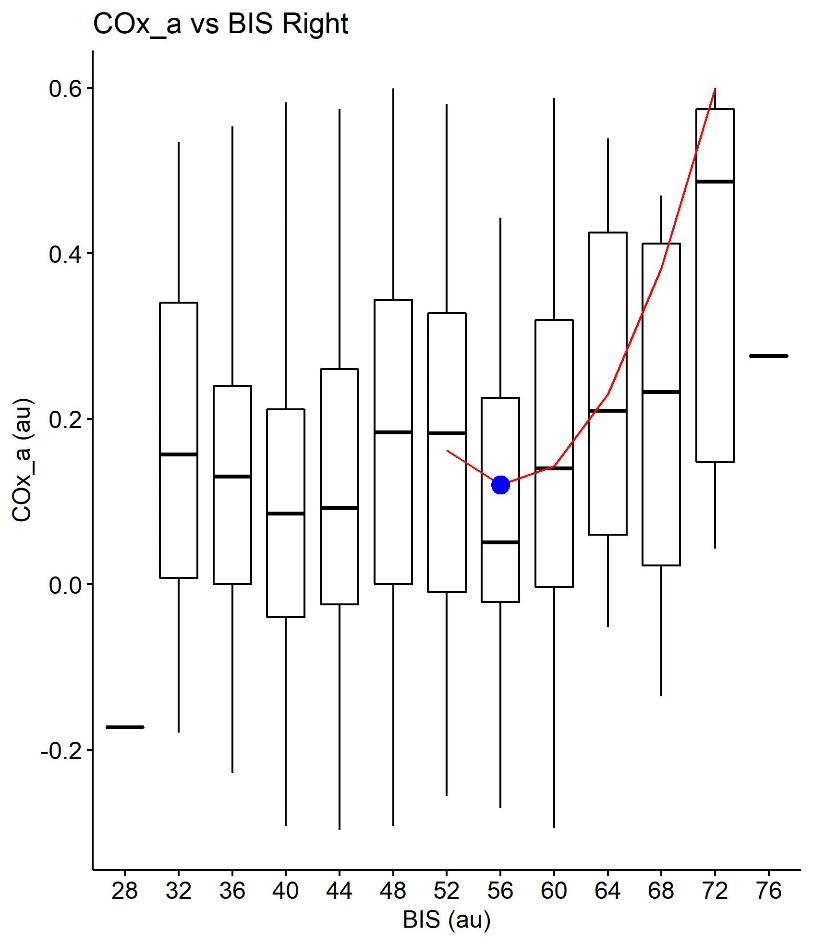


Patient 4 – U-Shaped Curves


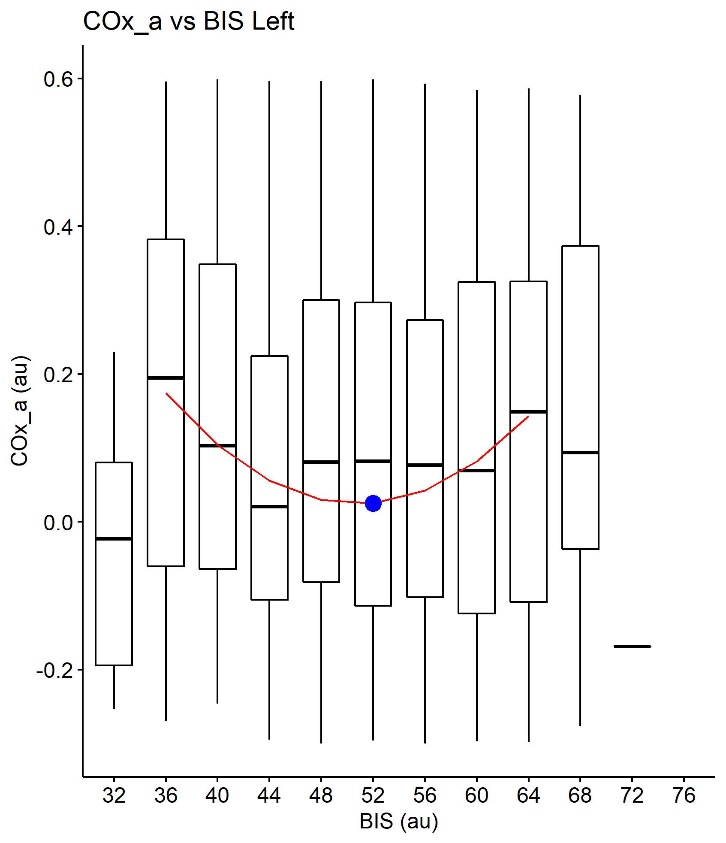

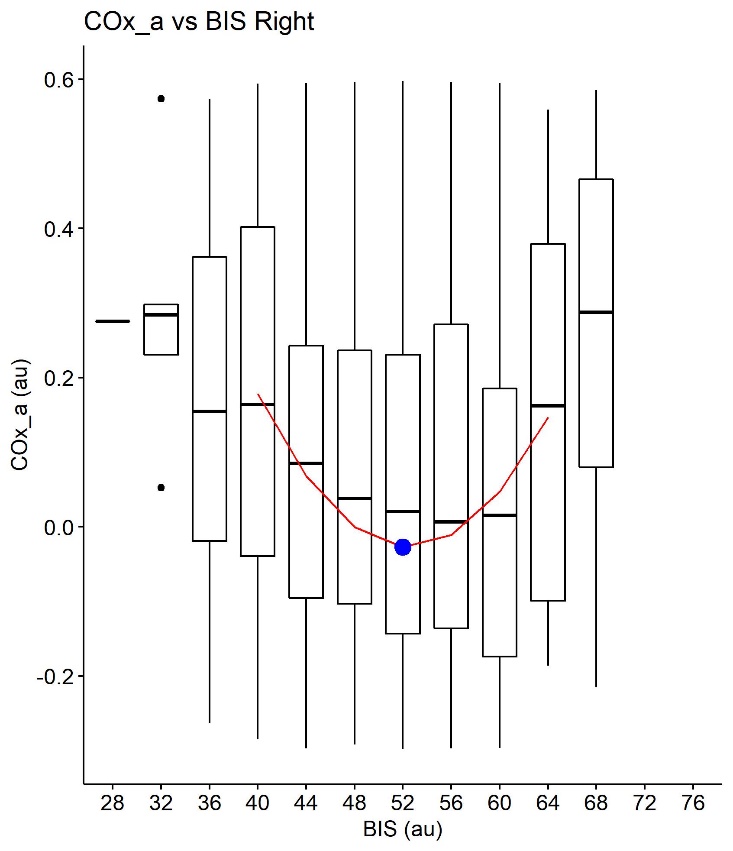


Patient 5 – Ascending Curve


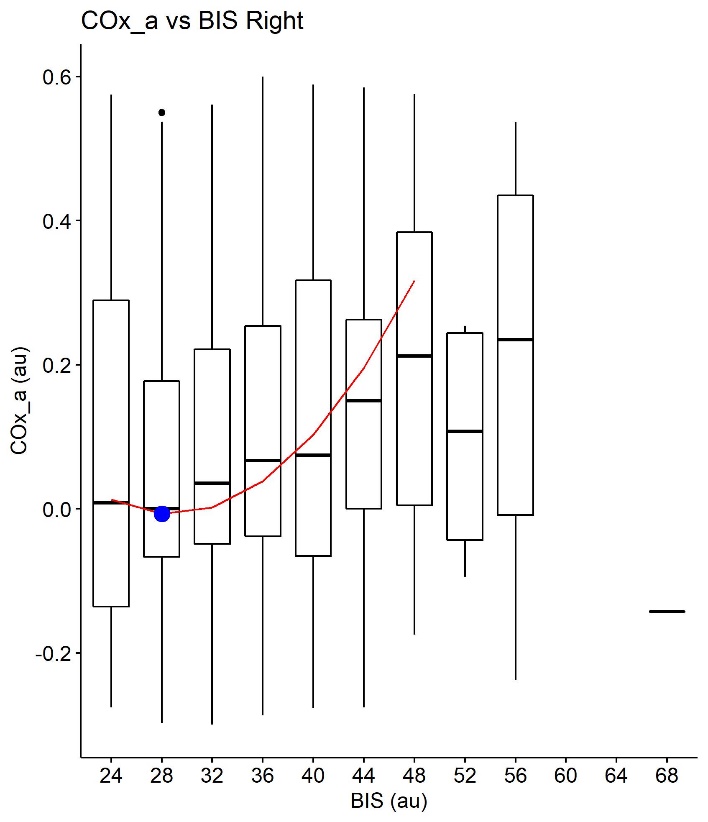


Patient 6 – Ascending Curve


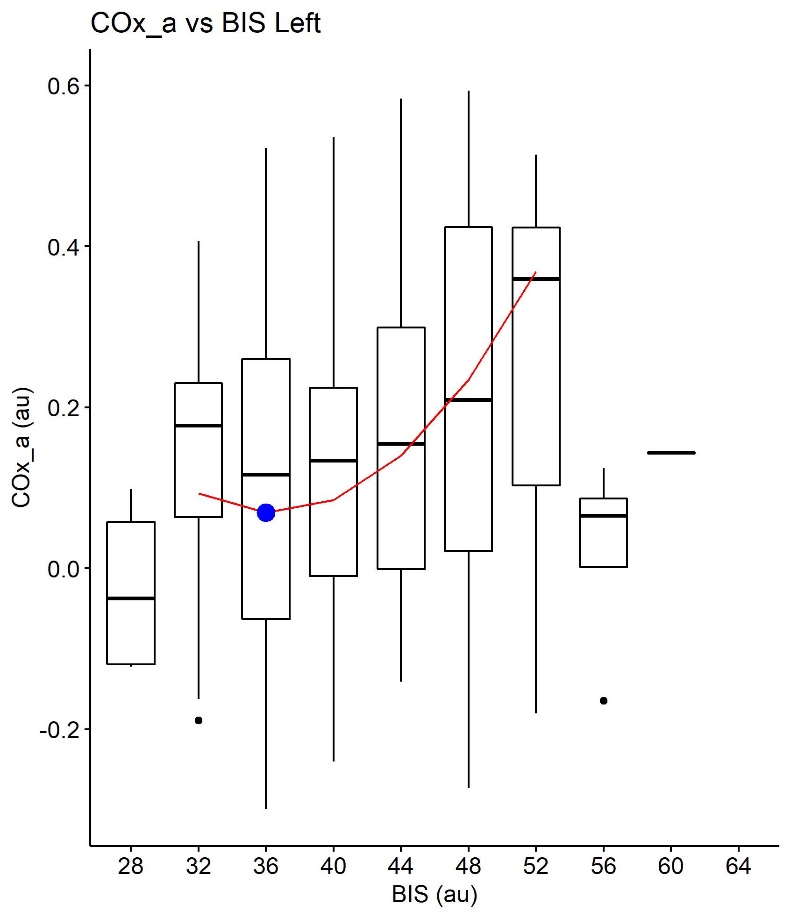


Patient 7 – U-Shaped Curves


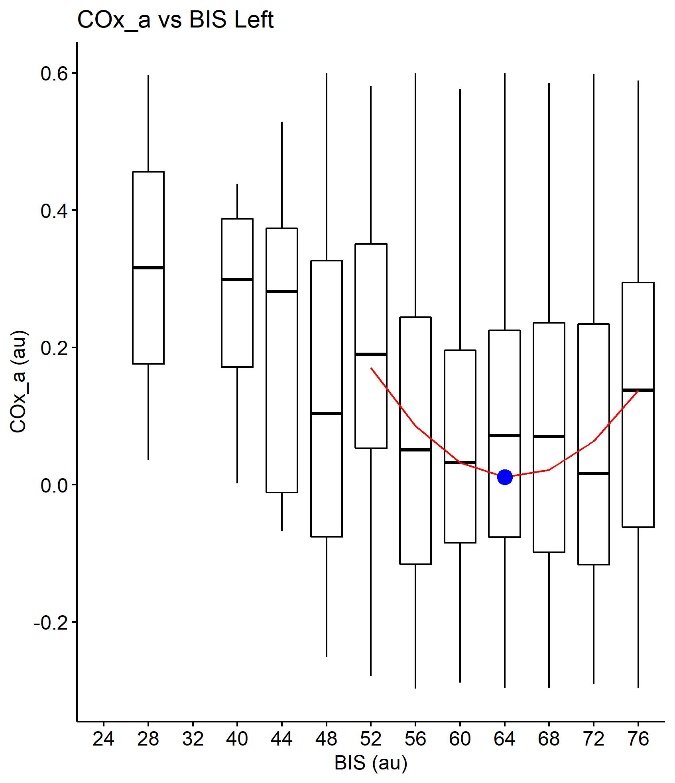

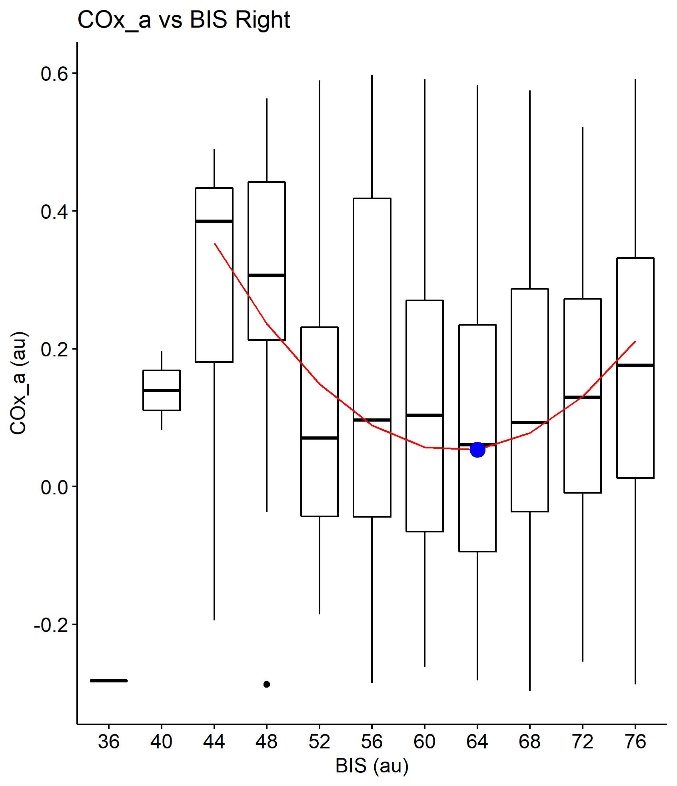


Patient 8 – U- Shaped Curve


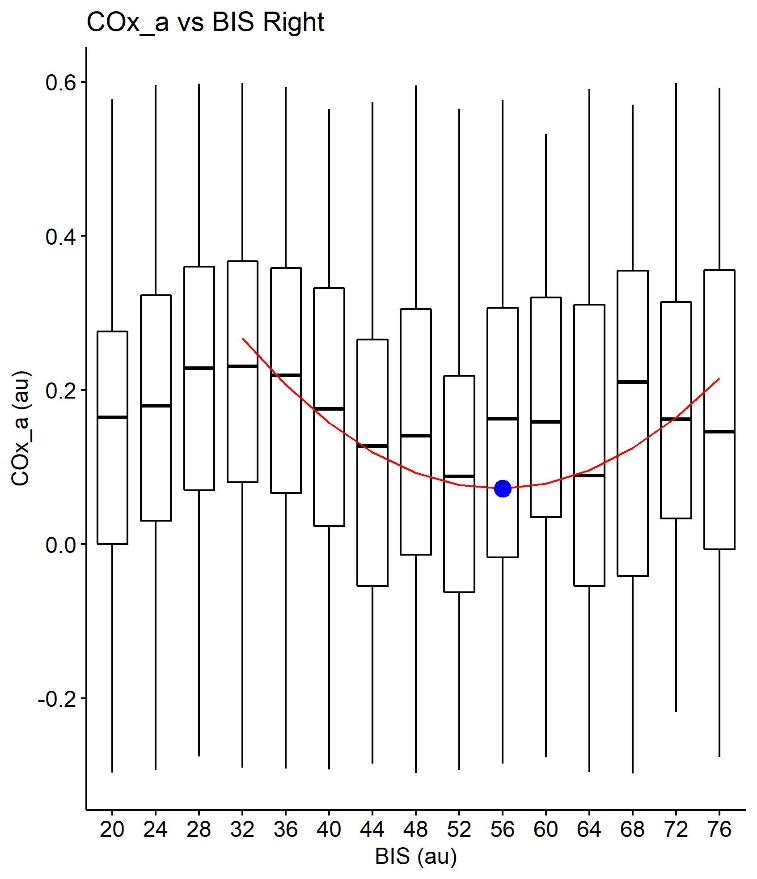


Patient 9 – Ascending Curves


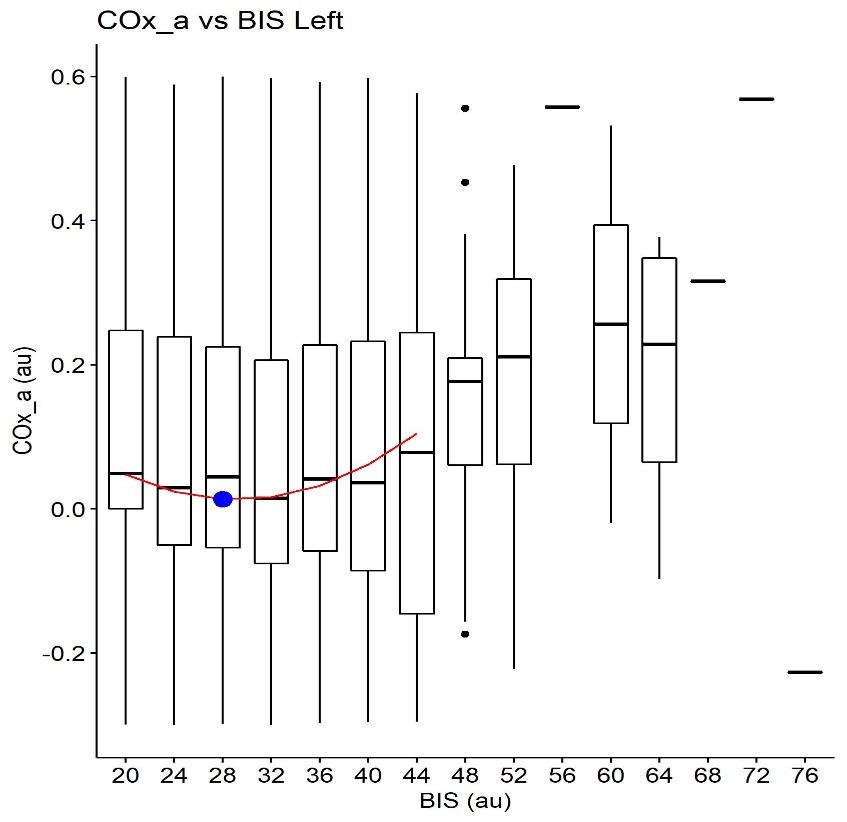

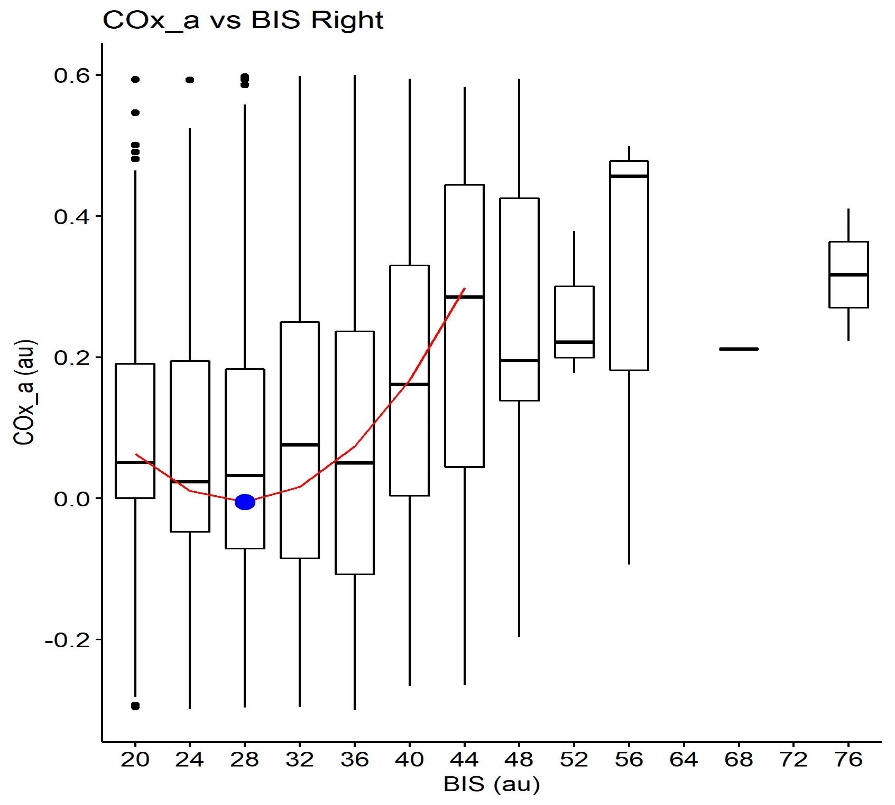


Patient 10 – U-Shaped Curve


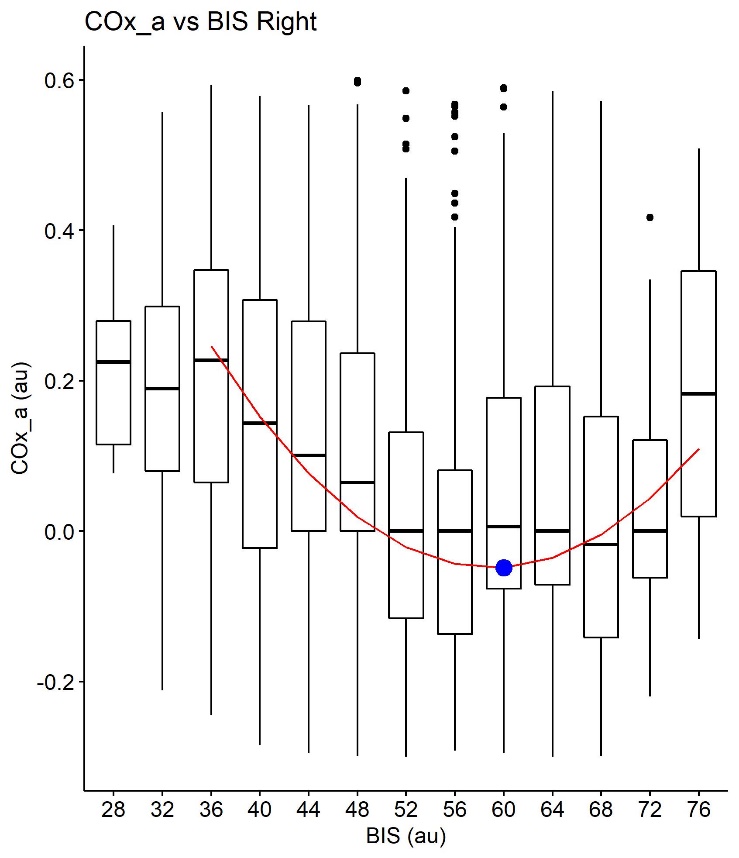


Patient 11 – U-Shaped Curves


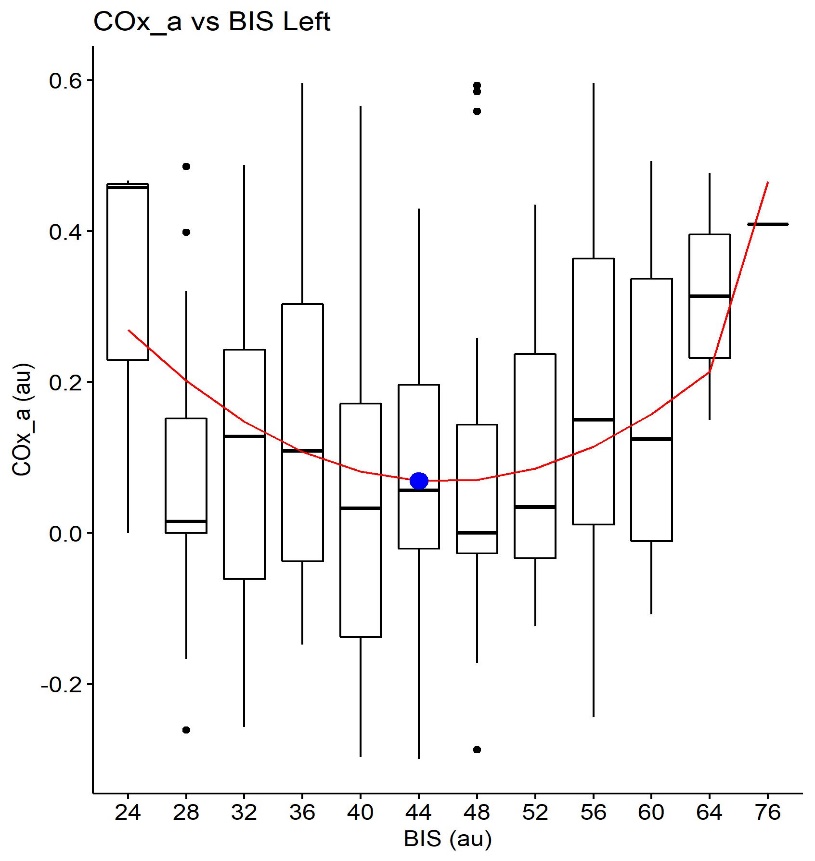

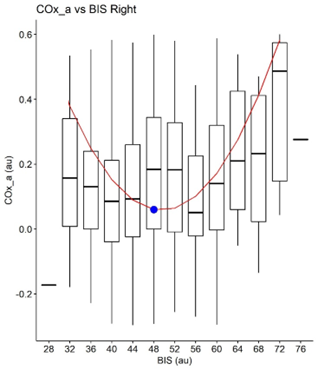


Patient 12 – U-Shaped Curve


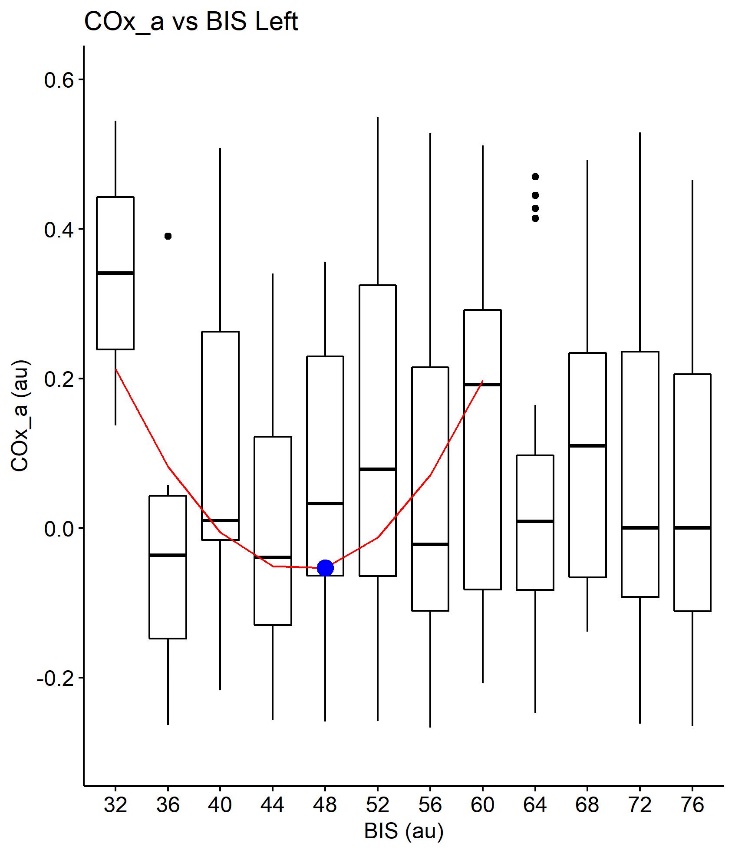


Patient 13 – Ascending Curves


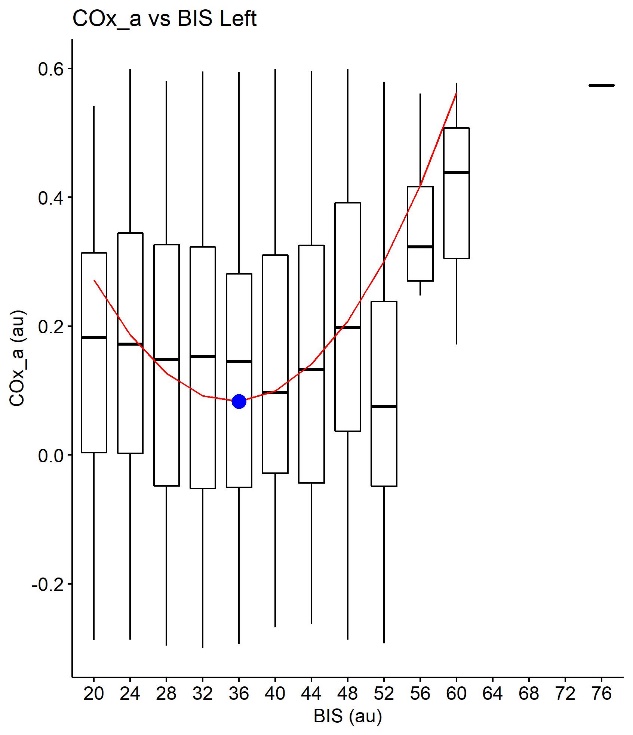

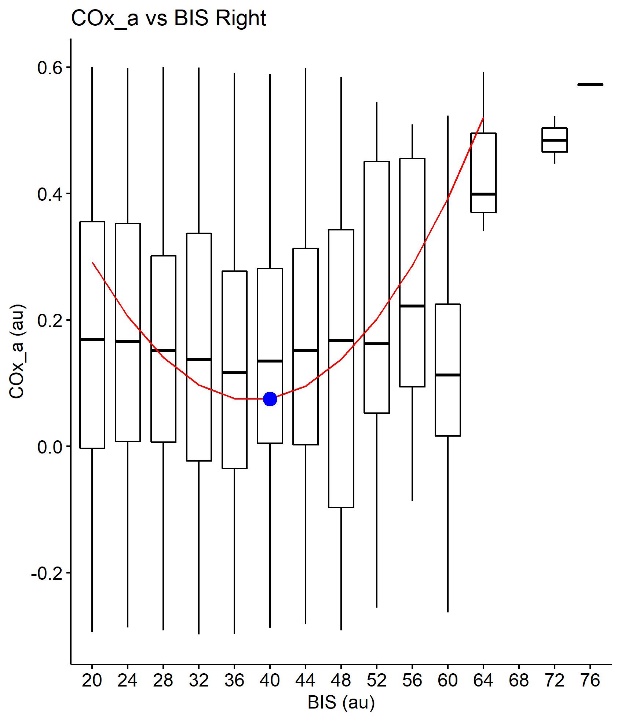


Patient 14 – U-Shaped Curve


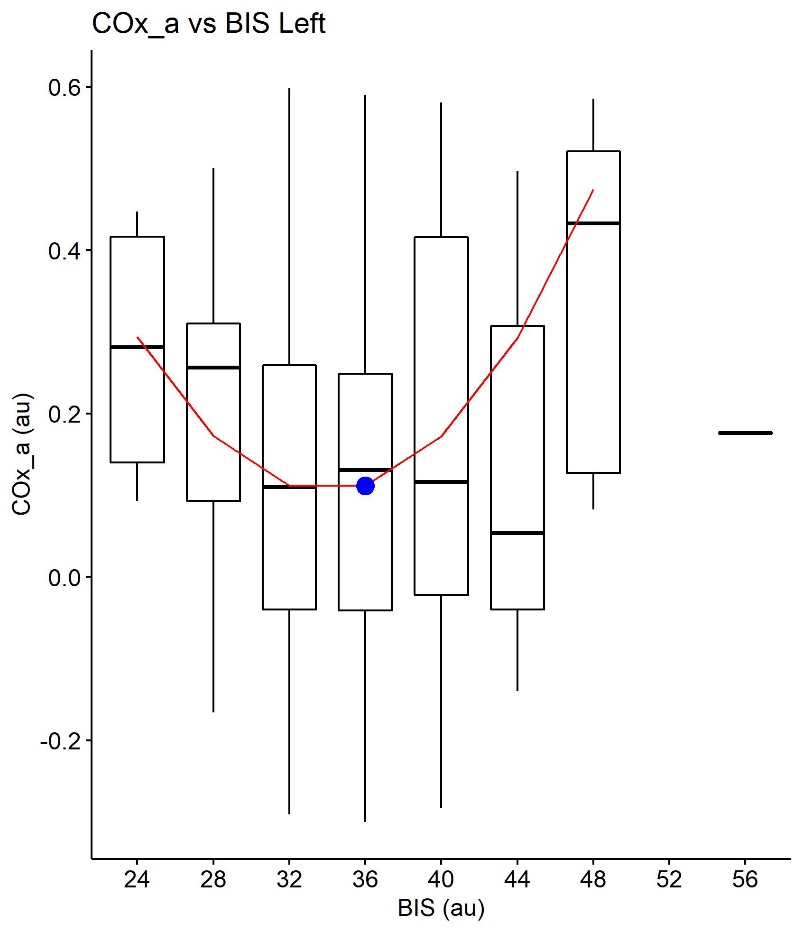


Patient 15 – U-Shaped Curve


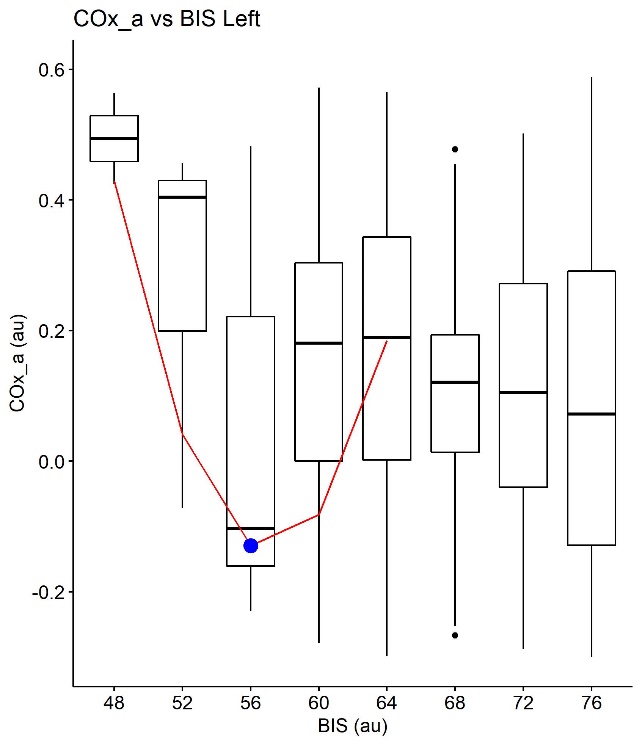


Patient 16 – U-Shaped Curve


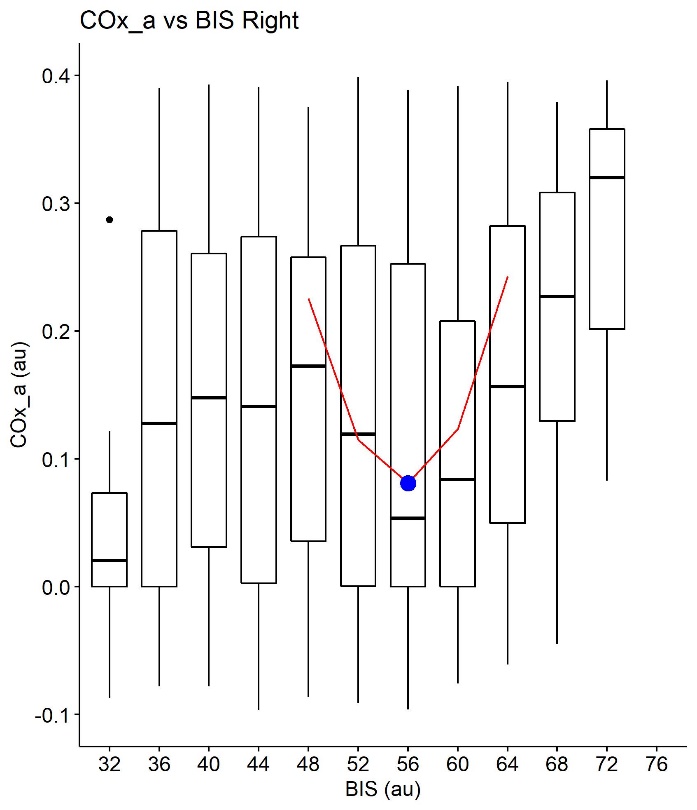


Patient 17 – U-Shaped Curve


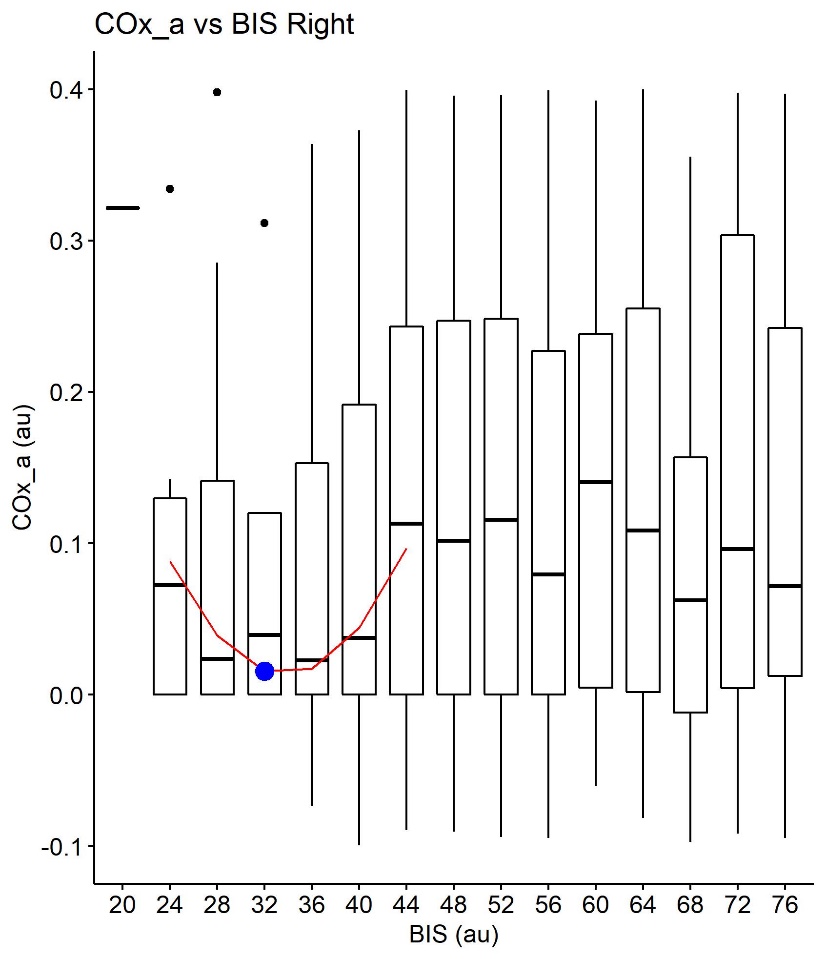


Patient 18 – Ascending Curve


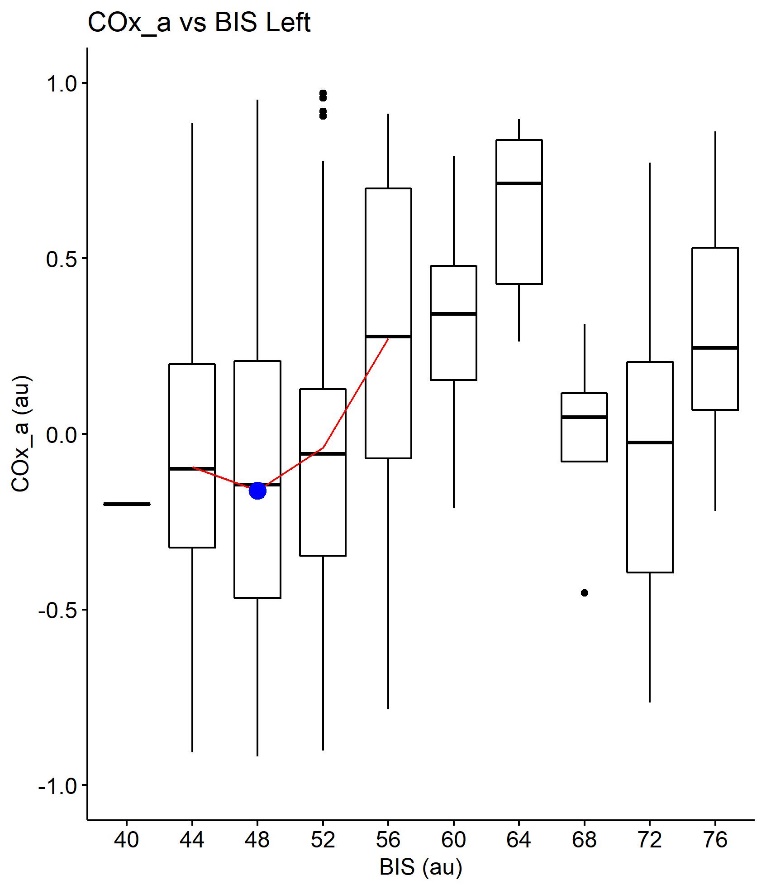


Patient 19 – Ascending Curve


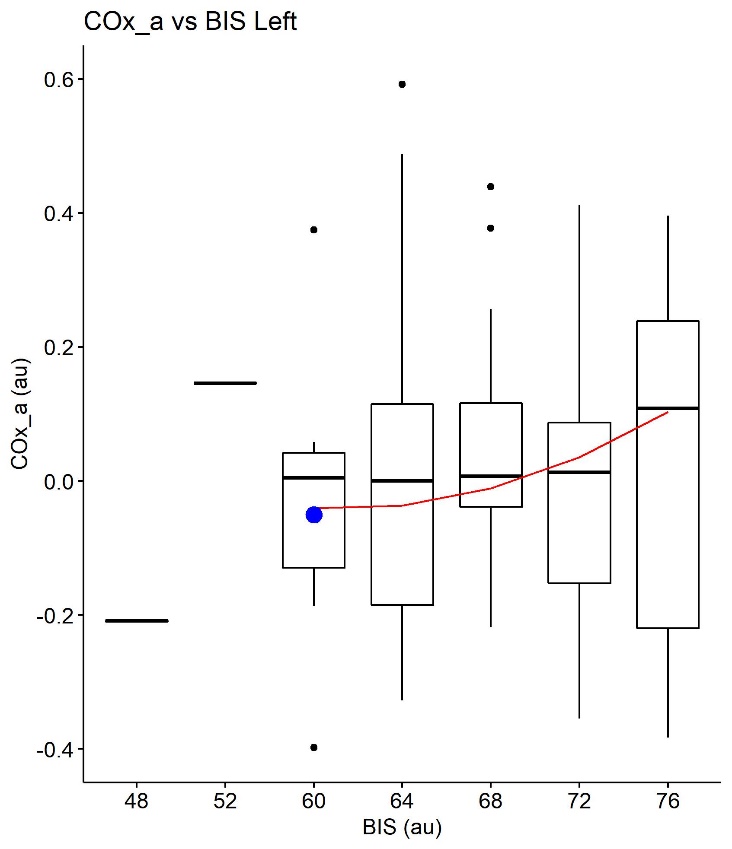


Patient 20 – U-Shaped Curve


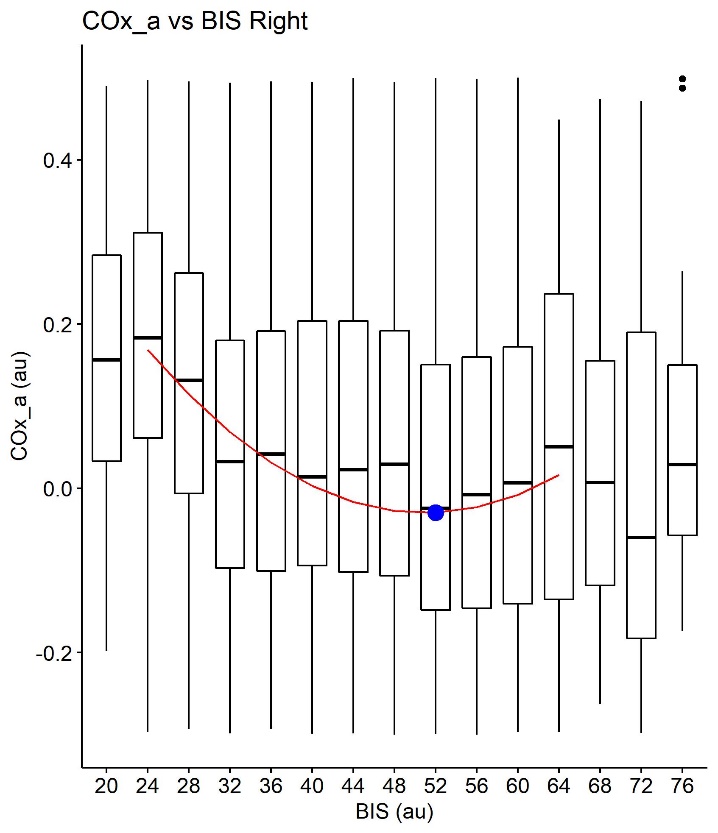


Patient 21 – Ascending Curve


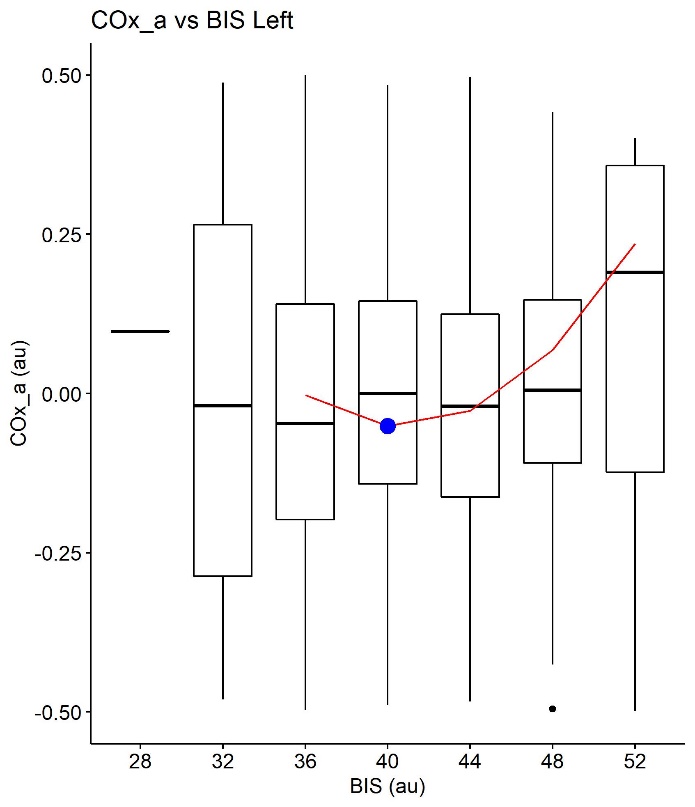


Patient 22 – U-Shaped and Ascending Curves


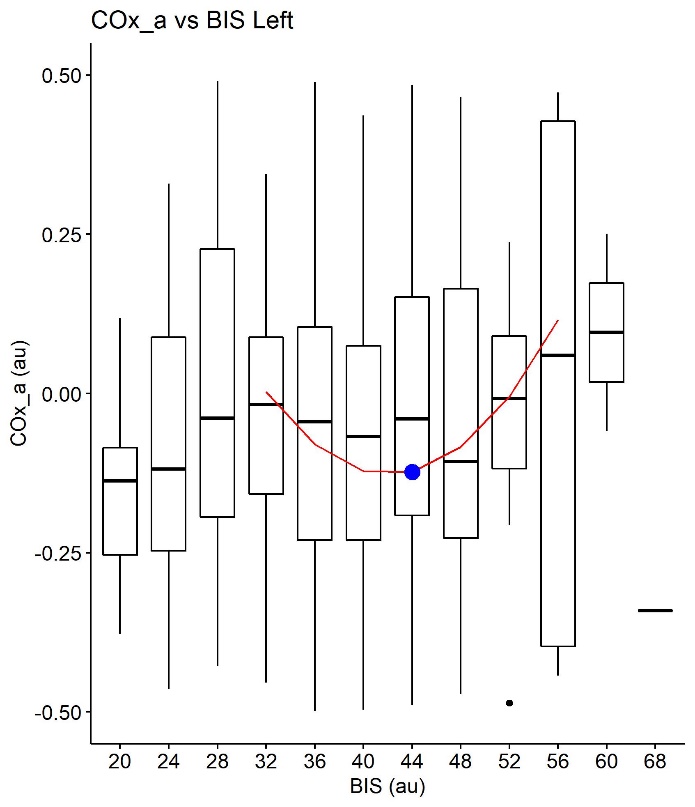

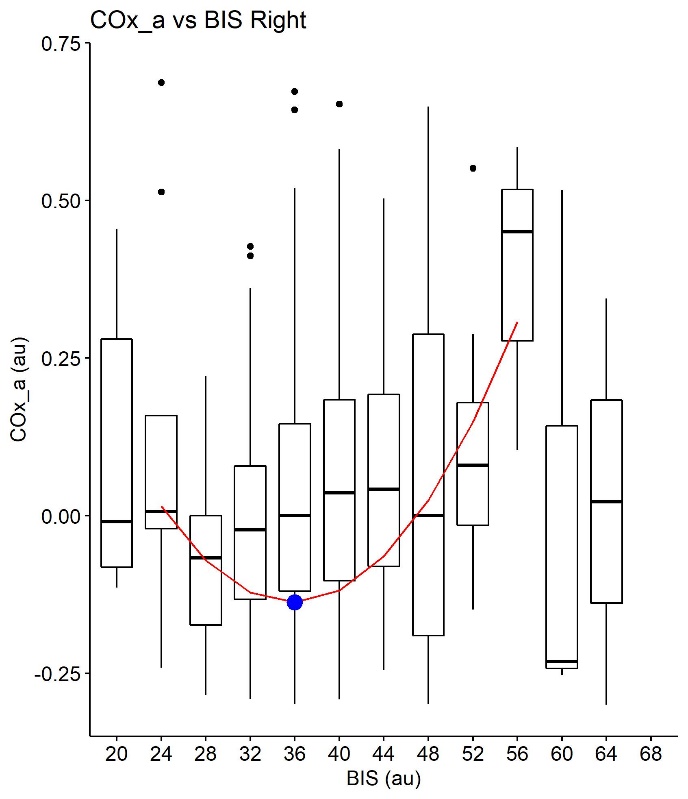


Patient 23 – Ascending Curve


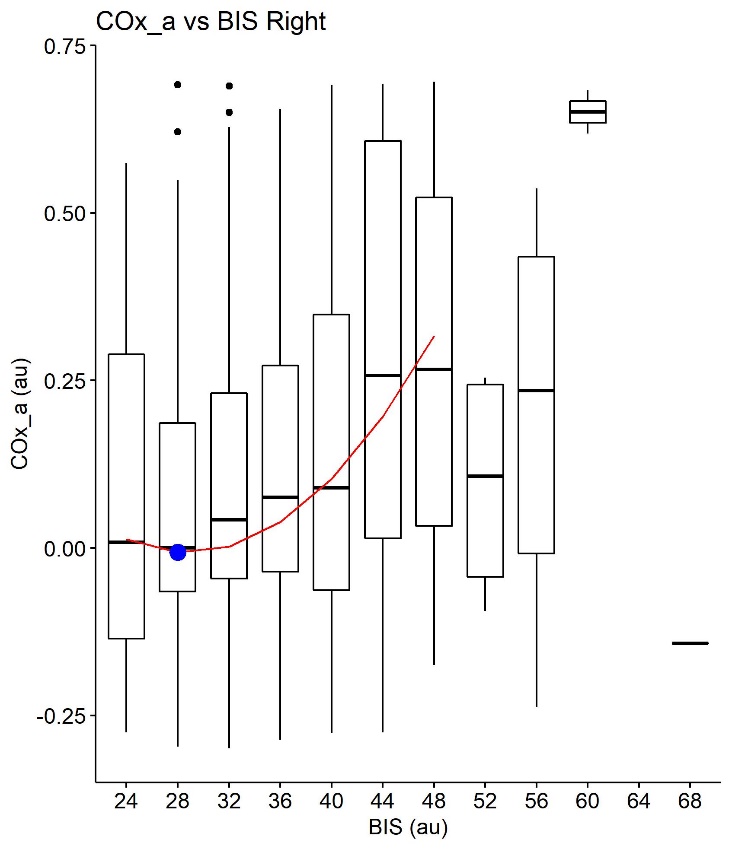


Patient 24 – U-Shaped Curves


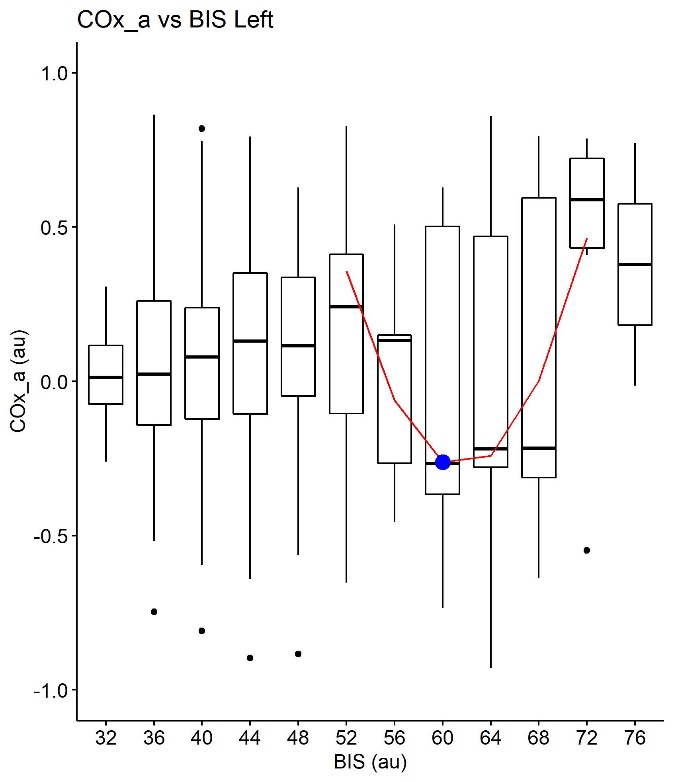

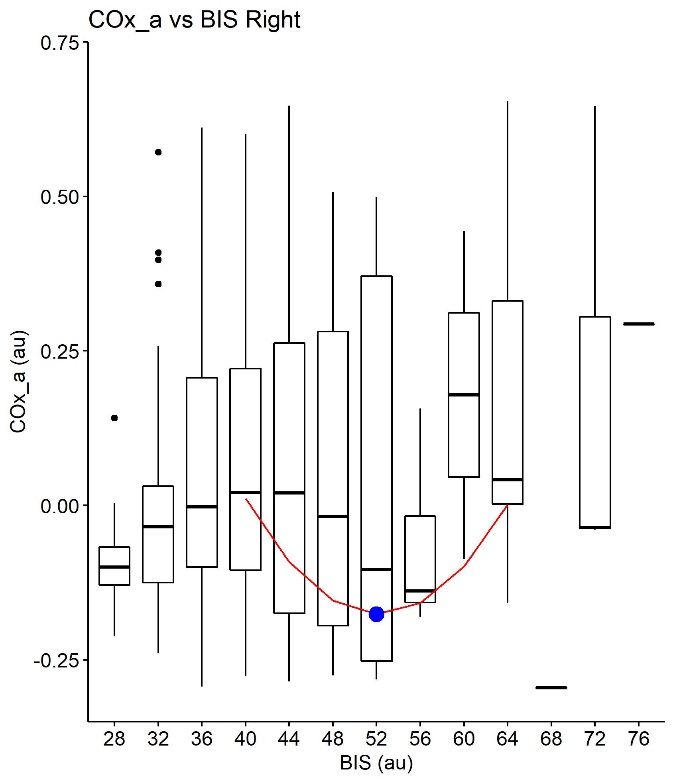


Patient 25 – U-Shaped Curves


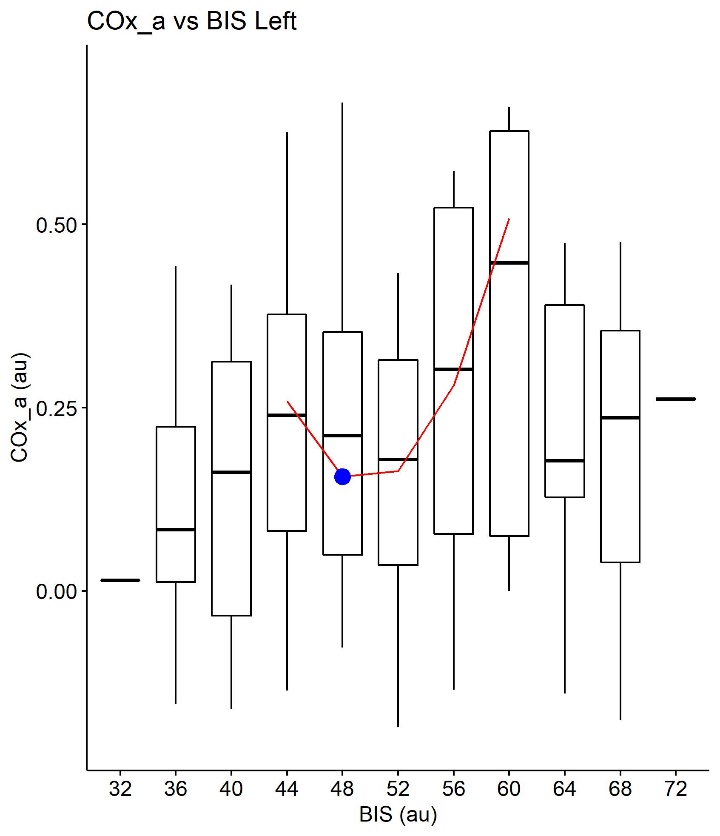

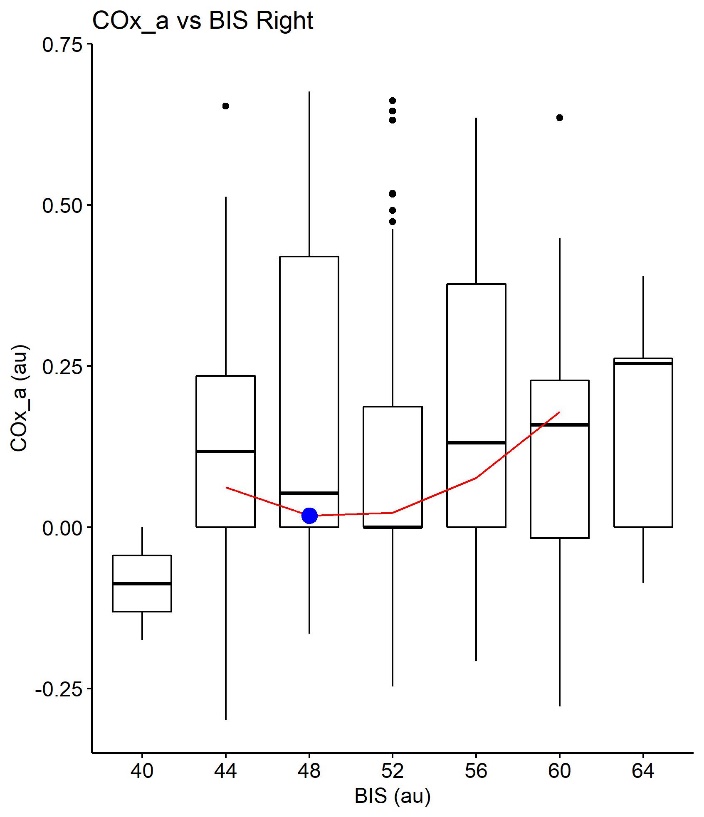


Patient 26 – U-Shaped Curve


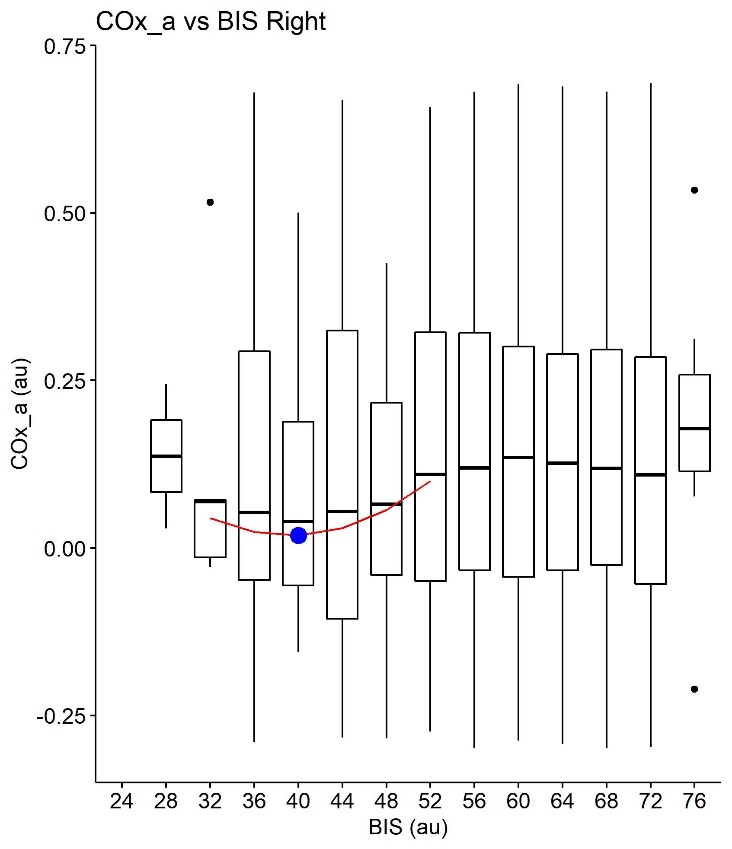


Patient 27 – U-Shaped Curve


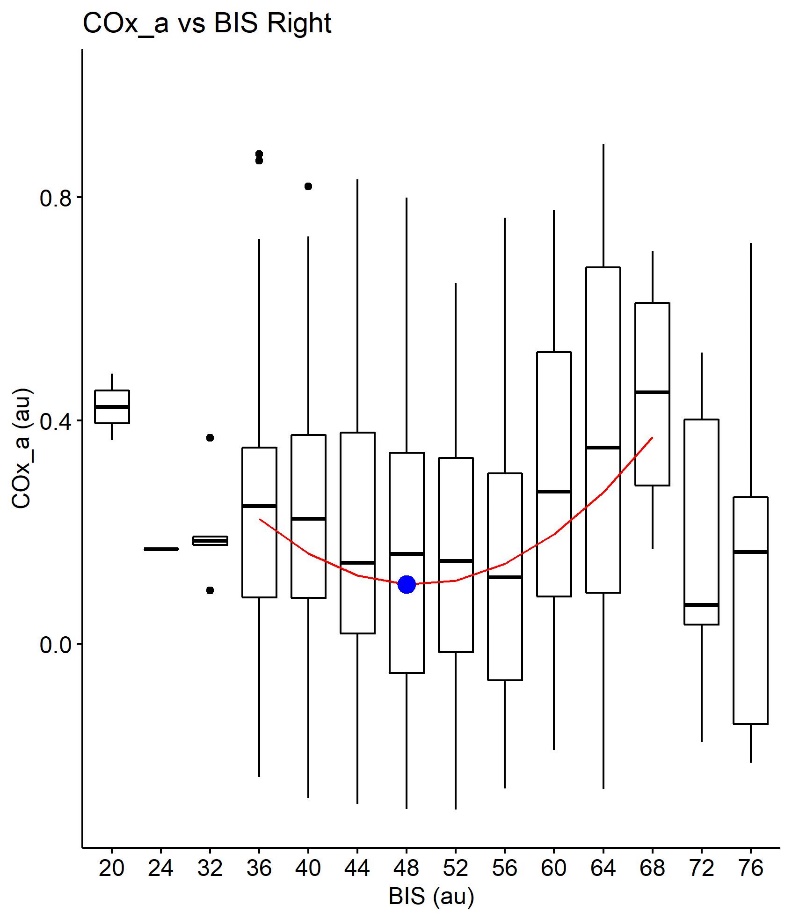


Patient 28 –U-Shaped Curve


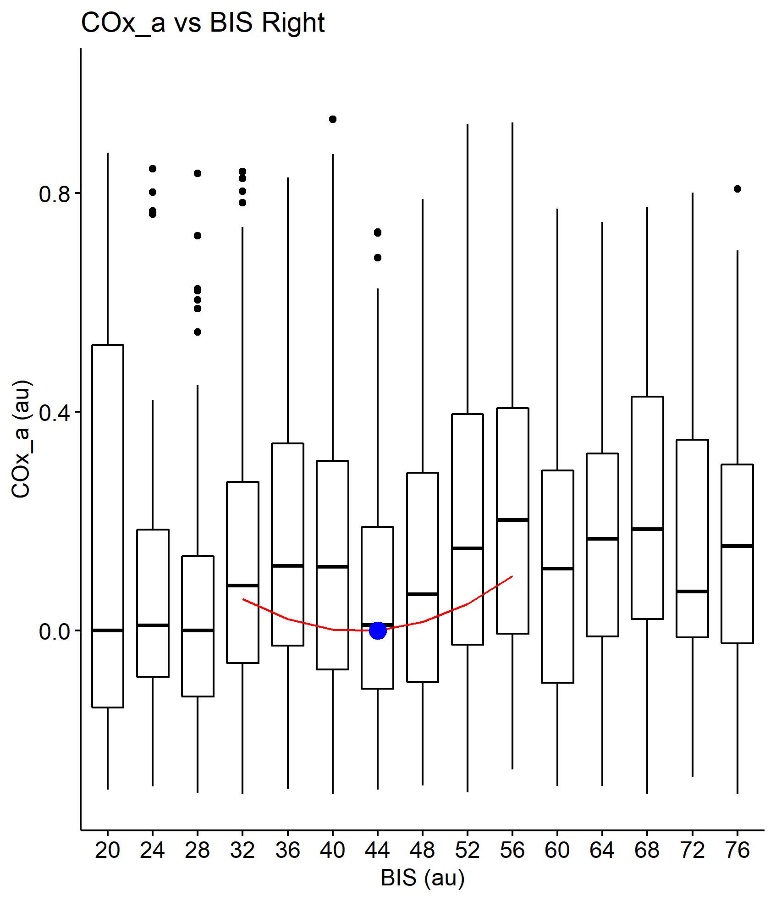


Patient 29 – U-Shaped Curves


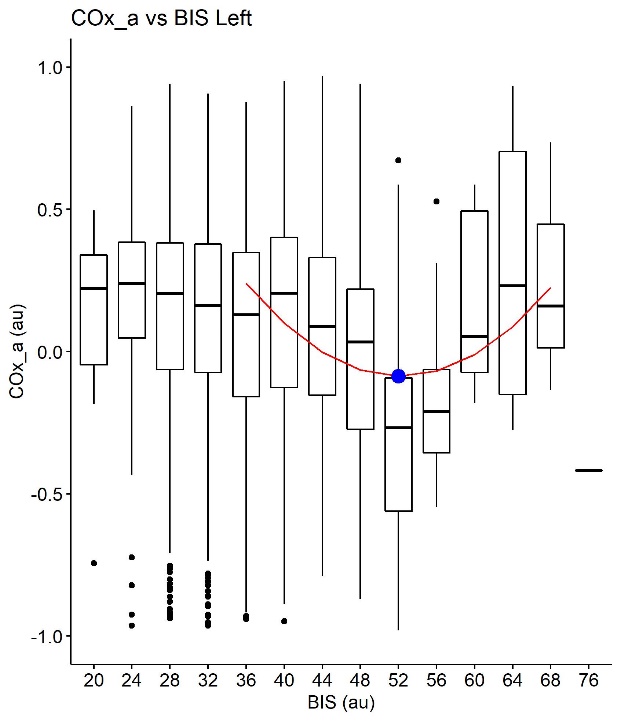

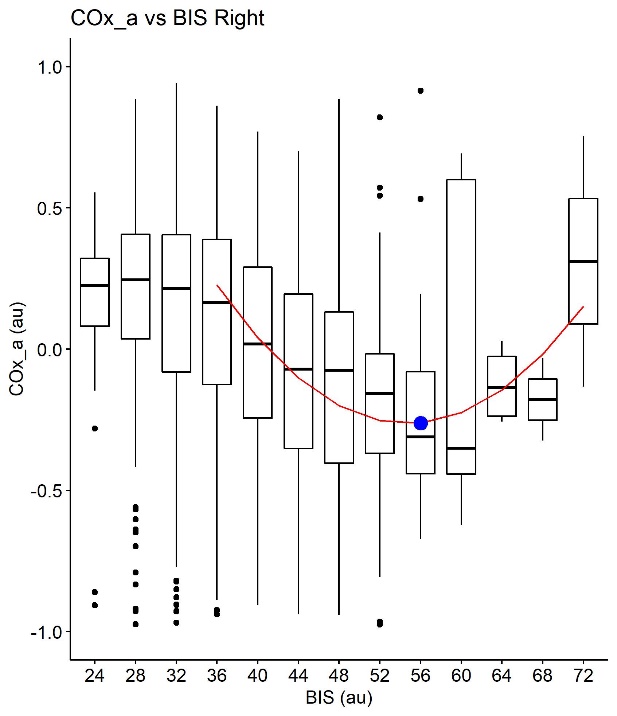


Patient 30 – U-Shaped Curves


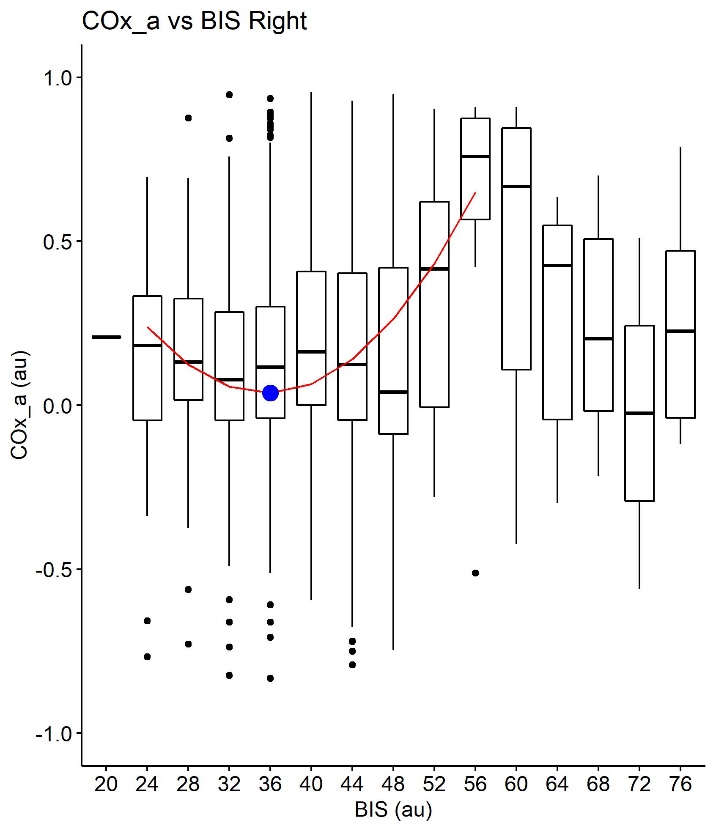


Patient 31 – Descending Curve


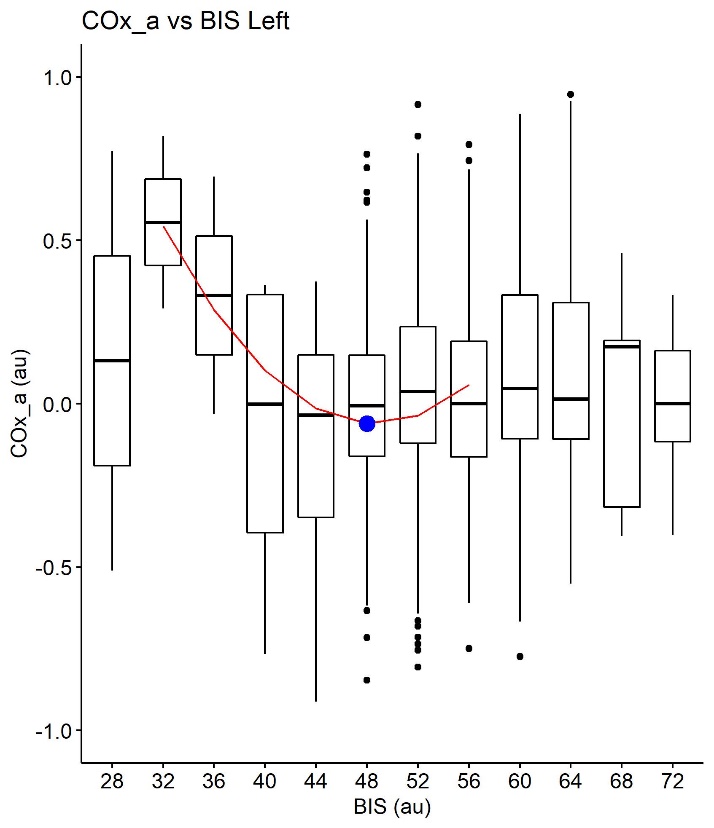


Patient 32 – U-Shaped Curve


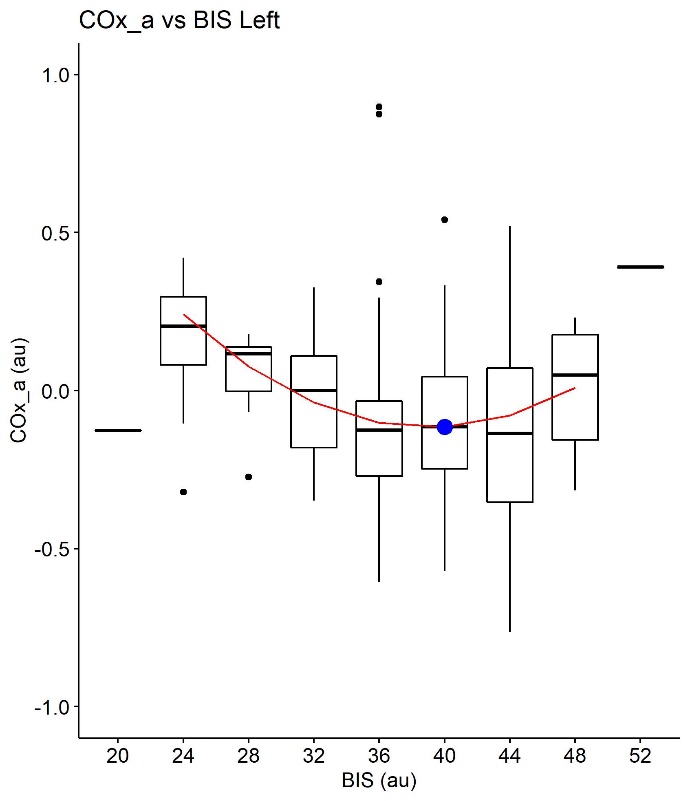


Patient 33 – Descending Curve


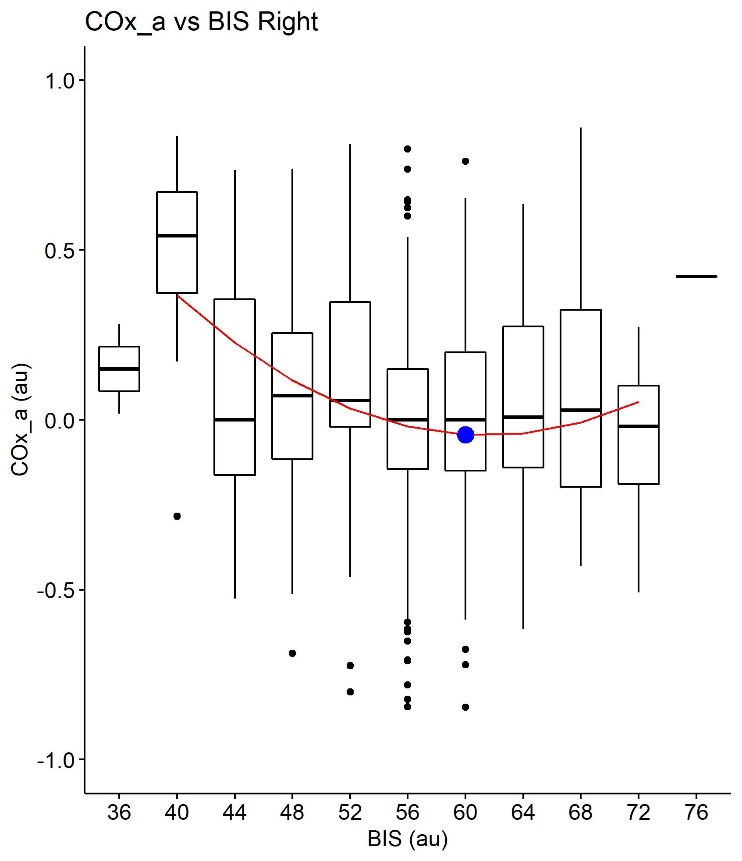

Supplement: Supplementary file 2 — Additional file 2. Supplementary Figures B. Examples of 33 Patient’s BISopt Derivation – Entire Recording. [file 40635_2022_460_MOESM2_ESM.docx]
