# Supplementary material for: Optimal bispectral index level of sedation and cerebral oximetry in traumatic brain injury: a non-invasive individualized approach in critical care?
Source: Intensive Care Med Exp. 2022 Aug 13;10:33. doi: 10.1186/s40635-022-00460-9 (PMC9375800; doi:10.1186/s40635-022-00460-9)

**Supplementary Figures C. Example of Patient with “Too Flat” Plot**

Boxplot represents the right or left side binned BIS values and the Fisher Z COx_a values. Note that this is an example of a “too flat” plot (COx_a change < 0.1). au, arbitrary units; BIS, bispectral index; COx_a, cerebral oximetry index.


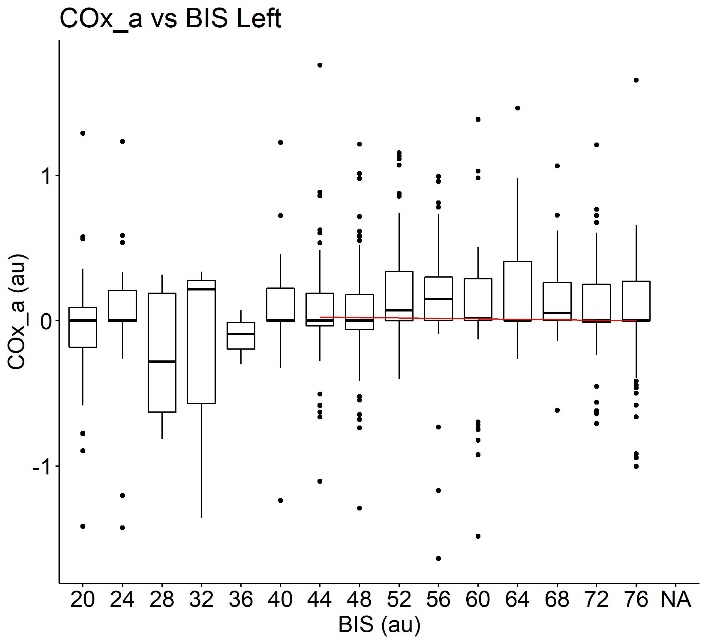

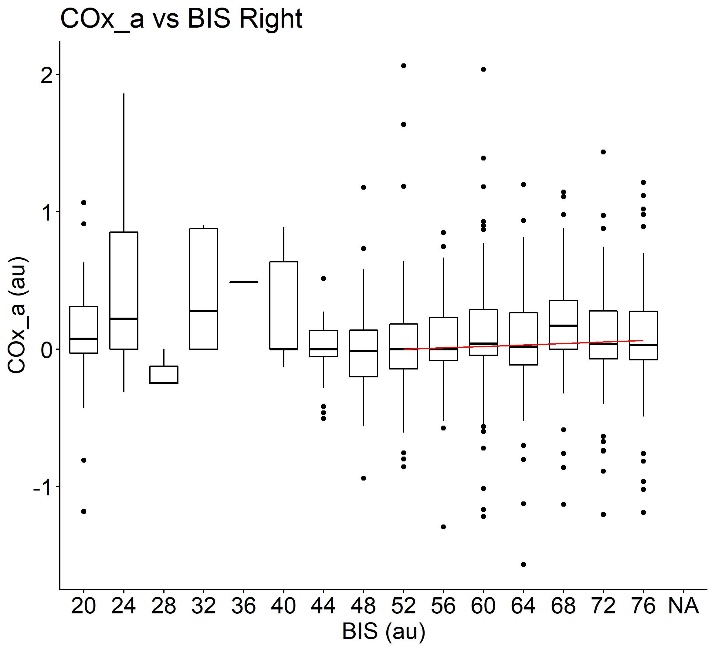

Supplement: Supplementary file 3 — Additional file 3. Supplementary Figures C. Example of Patient with “Too Flat” Plot. [file 40635_2022_460_MOESM3_ESM.docx]
