# Supplementary material for: Optimal bispectral index level of sedation and cerebral oximetry in traumatic brain injury: a non-invasive individualized approach in critical care?
Source: Intensive Care Med Exp. 2022 Aug 13;10:33. doi: 10.1186/s40635-022-00460-9 (PMC9375800; doi:10.1186/s40635-022-00460-9)

**Supplementary Figures D. Sequential Error Bar Plotting**

The analysis of one patient with 20 hours of continuous BIS and COx_a data. This allows the consistency of sequential error bar plotting using consecutive 4-hour windows. Thus, the analysis provides preliminary evidence of the potential to derive continuous optimal sedation depths using COx_a. au, arbitrary units; BIS, bispectral index; COx_a, Cerebral oximetry index

Figure D1. First 4 Hours


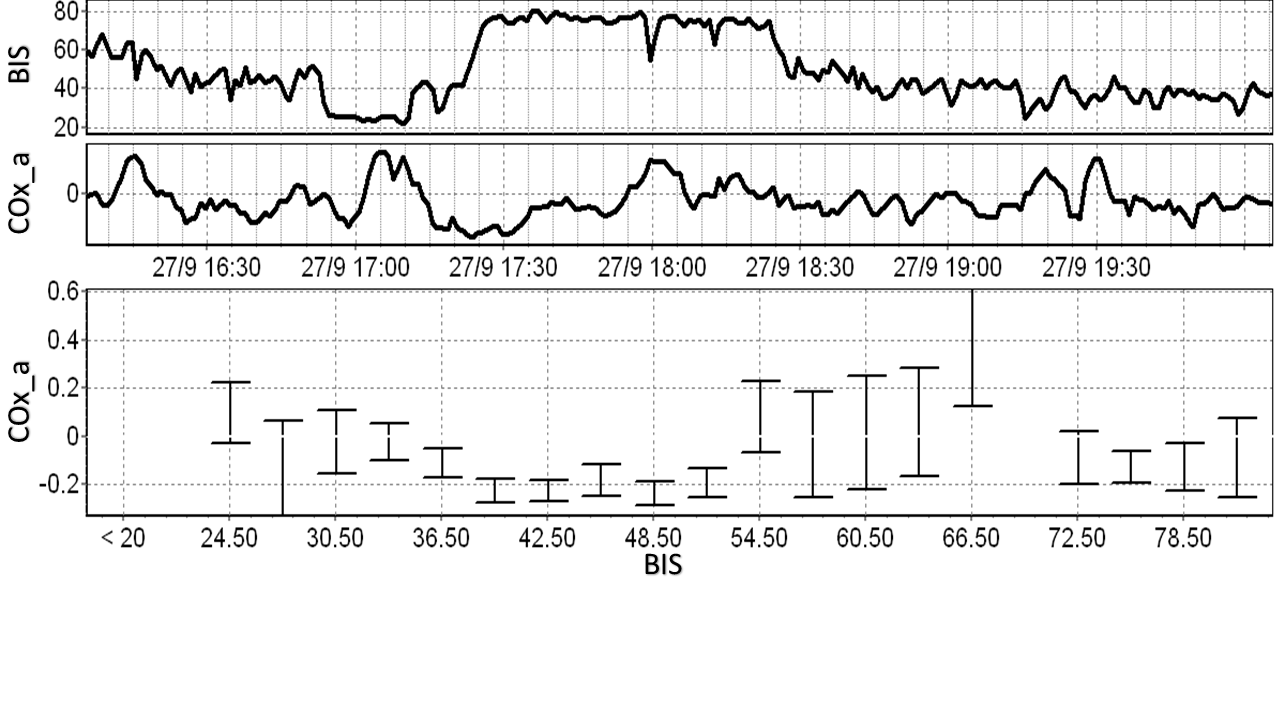


Figure D2. Second 4 Hours


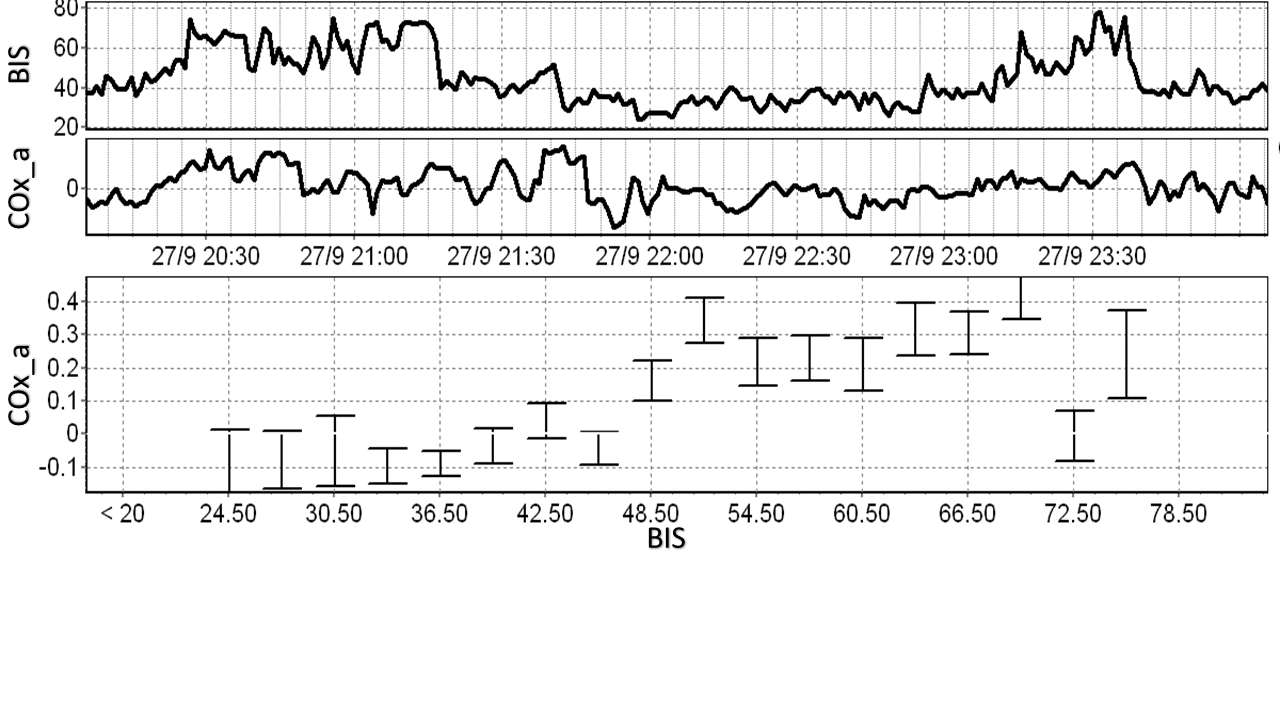


Figure D3. Third 4 Hours


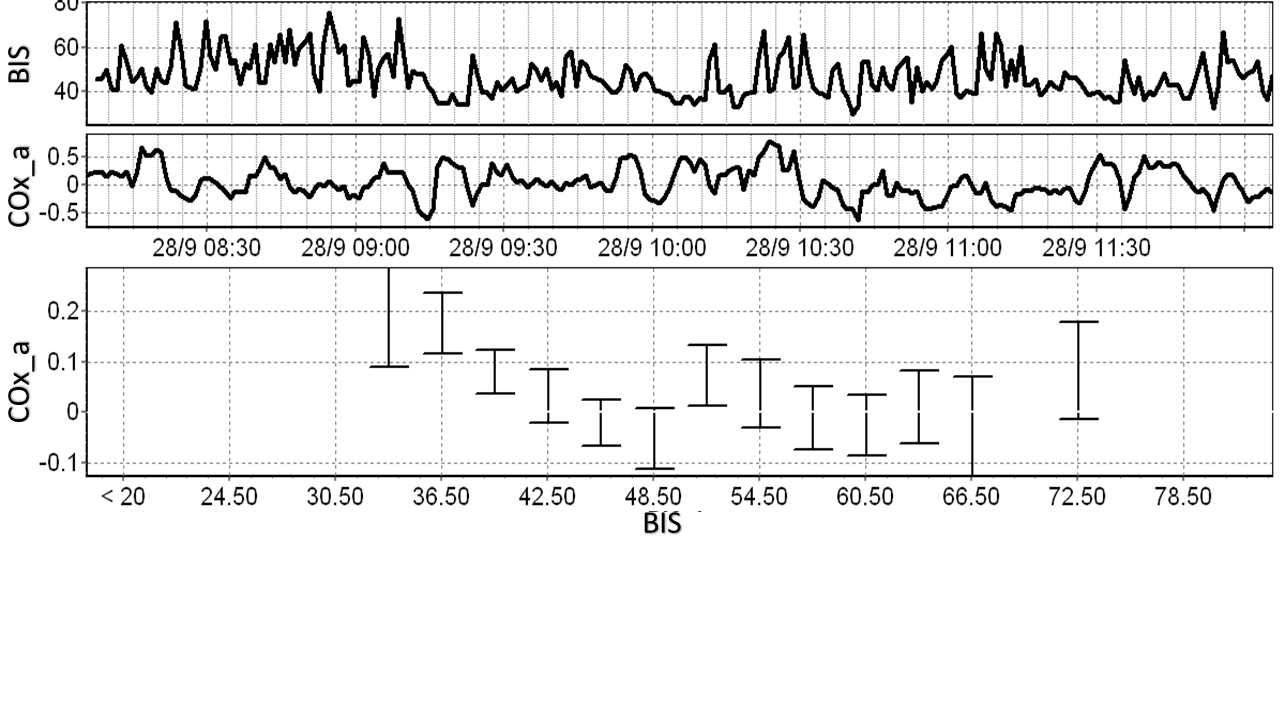


Figure D4. Forth 4 Hours


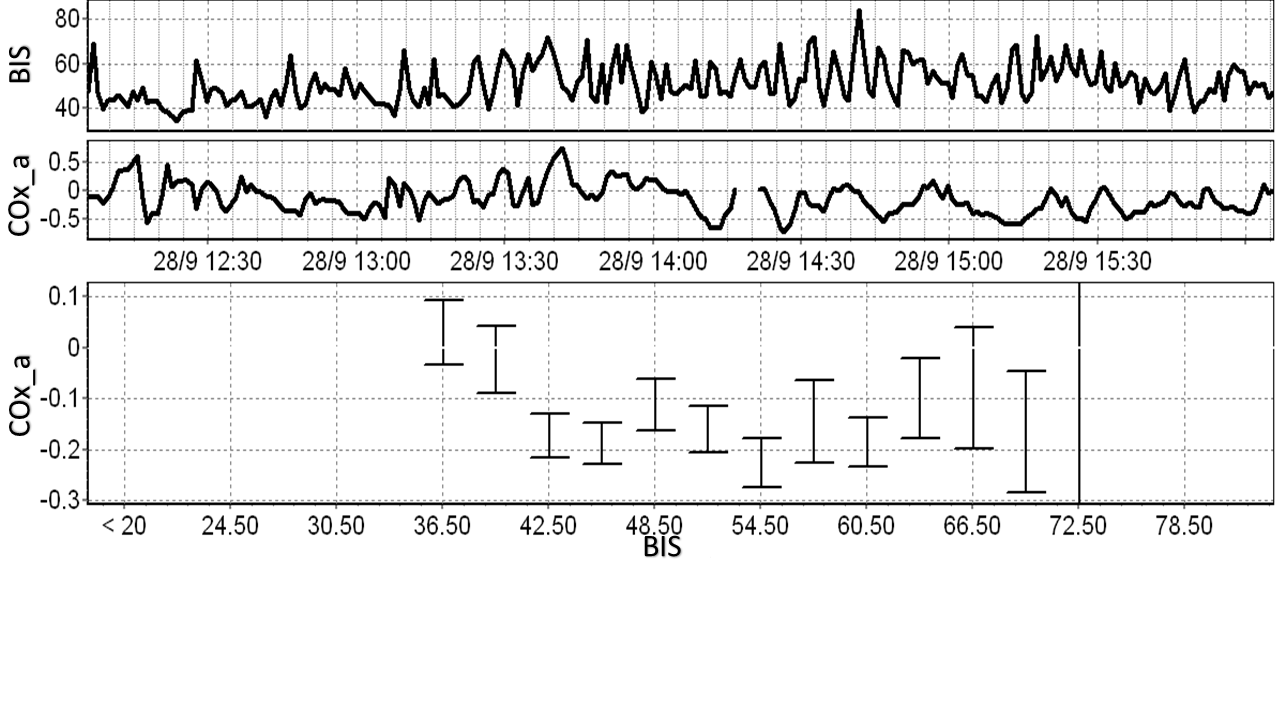

Supplement: Supplementary file 4 — Additional file 4. Supplementary Figures D. Sequential Error Bar Plotting. [file 40635_2022_460_MOESM4_ESM.docx]
