# Supplementary material for: Optimal bispectral index level of sedation and cerebral oximetry in traumatic brain injury: a non-invasive individualized approach in critical care?
Source: Intensive Care Med Exp. 2022 Aug 13;10:33. doi: 10.1186/s40635-022-00460-9 (PMC9375800; doi:10.1186/s40635-022-00460-9)
Supplement: Supplementary file 5 — Additional file 5. Supplementary Figures E. BISopt % Yeild. [file 40635_2022_460_MOESM5_ESM.docx]

**Supplementary Figures E. BISopt % Yeild**

Table E1 and E2 identifies the % yield and its association between sedative and vasopressor agents. This highlights the fact that BISopt appears to be calculable no matter the patient agent state.

Table E1. Agent Dose and BISopt % Yield

| Agent | Median %Yield (IQR) | Number of Patients | Median Recording Time (IQR) hrs. |
| --- | --- | --- | --- |
| Heavy Fentanyl (> 200 ug/kg/min) | 0.83 (0.452-0.915) | 3 | 6.733 (5.375-7.25) |
| Moderate Fentanyl (200-50 ug/kg/min) | 0.24 (0.163-0.289) | 7 | 59.6 (24.833-66.608) |
| Low Fentanyl (< 50 ug/kg/min) | 0.414 (0.073-0.559) | 20 | 13.508 (4.729-38.221) |
|  |  |  |  |
| Heavy Propofol (> 3 ug/kg/min) | 0.415 (0.208-0.623) | 2 | 3.933 (2.017-5.85) |
| Moderate Propofol (1-3 ug/kg/min) | 0.449 (0.195-0.562) | 28 | 6.758 (3.692-25.746) |
| Low Propofol (< 1 ug/kg/min) | 0.565 (0.221-0.836) | 24 | 7.442 (2.55-17.829) |

Table E2. Agent Type and BISopt % Yield

| Agent | Median %Yield (IQR) | Number of Patients | Median Recording Time (IQR) hrs. |
| --- | --- | --- | --- |
| Fentanyl | 0.444 (0.037-0.679) | 10 | 9.958 (6.771-14.171) |
| Propofol | 0.503 (0.257-0.684) | 19 | 12.45 (5.683-32.067) |
| Fentanyl and Propofol | 0.556 (0.263-0.746) | 15 | 5.3 (3.667-14.1) |
| Fentanyl and Ketamine | 0.117 (0.117-0.117) | 1 | 13 (13-13) |
| Fentanyl and Midazolam | 0.302 (0.218-0.306) | 3 | 68.017 (35.608-78.342) |
| Propofol and Midazolam | 0.314 (0.314-0.314) | 1 | 8.8 (8.8-8.8) |
| Fentanyl, Midazolam and Ketamine | 0.4 (0.273-0.617) | 4 | 46.225 (24.796-73.158) |
| Fentanyl, Propofol and Midazolam | 0.133 (0.084-0.436) | 5 | 19.15 (16.8-22.717) |
| Fentanyl, Propofol, Midazolam and Ketamine | 0.019 (0.01-0.029) | 2 | 23.3 (12.65-33.95) |
|  |  |  |  |
|  |  |  |  |
| Milrinone | 0.83 (0.83-0.83) | 1 | 7.767 (7.767-7.767) |
| Norepinephrine | 0.504 (0.339-0.76) | 27 | 7.7 (4.483-15.358) |
| Phenylephrine | 0.062 (0.062-0.062) | 1 | 14.667 (14.667-14.667) |
| Norepinephrine and Vasopressin | 0.32 (0.125-0.402) | 17 | 18.767 (9.817-43.65) |

*Tables for the median recording time and median % yield for a respective combination of agent or dose amount. All other combinations of sedative agents or vasopressor agents did not occur. Note that we did not separate the vasopressor agent and sedative agent group. BISopt, optimal bispectral index value; Hrs. hours; IQR, interquartile range; kg, kilogram; min, minute; ug, microgram;*
