# Supplementary material for: Optimal bispectral index level of sedation and cerebral oximetry in traumatic brain injury: a non-invasive individualized approach in critical care?
Source: Intensive Care Med Exp. 2022 Aug 13;10:33. doi: 10.1186/s40635-022-00460-9 (PMC9375800; doi:10.1186/s40635-022-00460-9)

**Supplementary Figures F. COx_a vs PRx BISopt Values (33 Patients)**

The figure demonstrates the patient that have both a BISopt value for COx_a and PRx over the whole data, and good U-shaped curve to identify the minimum value. For patients without an idealized U-shaped curve for both BISopt with PRx and COx_a, these BISopt values were omitted. The Wilcox-signed ranked test between the two tables is p= 0.31. 48 (IQR: 40-56) vs 45 (IQR: 40-56) for COx_a vs PRx BISopt. au, arbitrary units; COx_a, cerebral oximetry index; PRx, pressure reactivity index; BISopt, optimal sedation index.


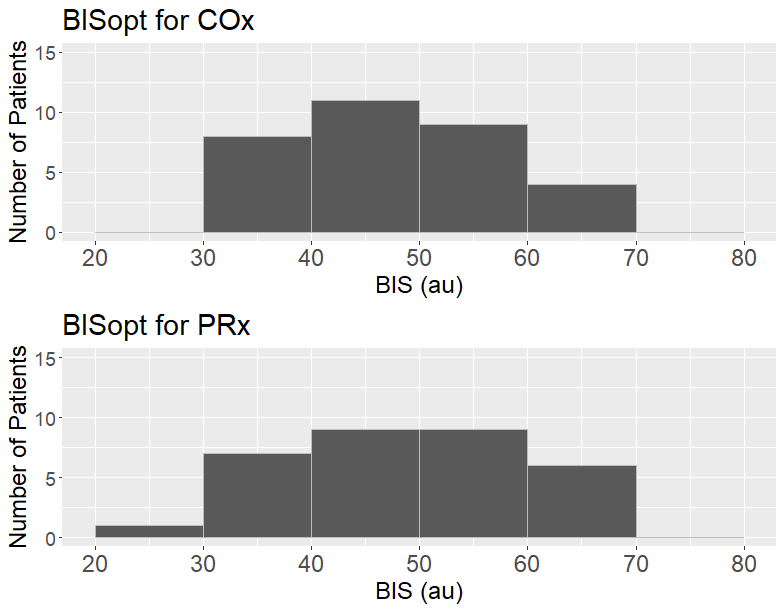

Supplement: Supplementary file 6 — Additional file 6. Supplementary Figures F. COx_a vs PRx BISopt Values (33 Patients). [file 40635_2022_460_MOESM6_ESM.docx]
